# Let the Cat out of the Bag: Popular Android IoT Apps under Security Scrutiny

**DOI:** 10.3390/s22020513

**Published:** 2022-01-10

**Authors:** Efstratios Chatzoglou, Georgios Kambourakis, Christos Smiliotopoulos

**Affiliations:** 1 Department of Information & Communication Systems Engineering, University of the Aegean, 811 00 Mitilini, Greece; efchatzoglou@gmail.com (E.C.); chr.smiliotopoulos@gmail.com (C.S.); 2 European Union, Joint Research Centre, 21027 Ispra, Italy

**Keywords:** IoT, vulnerabilities, weaknesses, security, Android, static, dynamic

## Abstract

The impact that IoT technologies have on our everyday life is indisputable. Wearables, smart appliances, lighting, security controls, and others make our life simpler and more comfortable. For the sake of easy monitoring and administration, such devices are typically accompanied by smartphone apps, which are becoming increasingly popular, and sometimes are even required to operate the device. Nevertheless, the use of such apps may indirectly magnify the attack surface of the IoT device itself and expose the end-user to security and privacy breaches. Therefore, a key question arises: do these apps curtail their functionality to the minimum needed, and additionally, are they secure against known vulnerabilities and flaws? In seek of concrete answers to the aforesaid question, this work scrutinizes more than forty chart-topping Android official apps belonging to six diverse mainstream categories of IoT devices. We attentively analyse each app statically, and almost half of them dynamically, after pairing them with real-life IoT devices. The results collected span several axes, namely sensitive permissions, misconfigurations, weaknesses, vulnerabilities, and other issues, including trackers, manifest data, shared software, and more. The short answer to the posed question is that the majority of such apps still remain susceptible to a range of security and privacy issues, which in turn, and at least to a significant degree, reflects the general proclivity in this ecosystem.

## 1. Introduction

The mushrooming of Internet of Things (IoT) devices augments the security stakes and challenges by far, even to a degree that threatens the entire Internet community. By operating networks of myriads of IoT devices, cyber assailants can target through their Denial of Service (DoS) bomb sight any website or Internet service and potentially bring it down to its knees. Nowadays, the great majority of personal IoT devices, including Small Office Home Office (SOHO) ones such as wireless Access Points (APs) are accompanied by a smartphone application (app). In this respect, any potential security and privacy issue may stem from either the IoT device itself, the associated app, or both. Simply put, the attack surface of virtually any IoT device is conceivably increased due to the accompanying app. Furthermore, of course there exists a plethora of apps that can be used to control and administer a variety of devices and across disparate manufacturers or vendors.

Under this prism, it is of great interest to examine if and to what degree chart-topping mobile apps destined to IoT devices of diverse categories, say, smart TV, personal assistants, smart wearable, and others present security and privacy issues that can degrade the end-user’s security or privacy level, and directly or indirectly expose the device to attacks and hijacking. Particularly, we pursue to answer two key questions regarding the security and privacy state of this kind of apps: Do they restrict their functionality to the absolute minimum? and do they remain free of known misconfigurations, vulnerabilities, and privacy leaks?

Having this goal in mind, the work at hand scrutinizes more than 40 popular Android accompanying apps belonging to 6 different categories of IoT devices, namely APs, smart TVs, wireless IP cameras, smart wearable, smart assistant, and Smart bulb/plug. The examined apps are the official versions, that is, those provided by the respective vendor and uploaded to Google Play Store. The term “popular” means that we only consider apps that exceed 1M downloads.

The contributions of this work vis-à-vis the related work presented in Section 6 are as follows.

- We conduct an up-to-date, full-fledged, both shallow and deep analysis of more than four tens of mainstream IoT official Android apps spread across the 6 most popular diverse categories of home/office and wearable devices.
- Contrary to previous work that concentrates on device’s firmware, the use of fuzz testing, and individual app features, say, network communications, the results of our analysis stem from a plethora of both static and dynamic features, the latter after pairing the app with real-life well-known assorted IoT devices. In this respect, the methodology and the results given by this work tackle the specific issue from a more holistic viewpoint.
- The multi-category approach followed, caters for the drawing of important conclusions, directly originating from the juxtaposition of the distinct categories of apps.

Overall, the results provided by this work can at minimum serve a couple of objectives. The first is related to the improvement of the sound design of future IoT apps from a security and end-user privacy perspective. The audience here is mostly app designers and developers. Secondly, having in mind that the outcomes of this effort mostly reflect the general tendency in this field, it can be directly used as a basis towards advancing research efforts, mostly focusing on fostering best practices that facilitate the abatement of the attack surface, deterring data leakage, and cultivating a security and privacy by design mindset overall. In particular, for privacy, the key matter being in jeopardy here is the so-called principle of minimal privilege, requiring that a user, process, or piece of code in general, must be inherently enabled to only access the data and resources that are absolutely essential for accomplishing its mission.

The remainder of this paper is structured as follows. The next section briefly describes the methodology used to analyze the different apps. Section 3 elaborates on the Android permissions used by each app, focusing on sensitive runtime permissions from an end-user’s privacy perspective. Section 4 proceeds to a deeper level of static analysis by examining security and privacy issues related to the app’s code, the use of trackers, shared libraries, outdated libraries, and others. The results of dynamically analyzing several apps after pairing them with real-life IoT devices are given in Section 5. The related work is presented in Section 6. The last section concludes and gives pointers to future work.

## 2. Methodology

Forty-one apps (packages) were collected from the Google Play Store with data freeze as of 5 October 2021. The apps were chosen based on three distinct criteria, as follows.

- Based on the number of IoT devices we possess, which naturally is bounded, six popular IoT categories were created, namely, APs, smart TV, wireless IP cameras, smart wearable, smart assistant, and smart bulb/plugs. In this sense, IoT devices that fall into other IoT device categories, say, smart home climate control appliances or smart locks, were intentionally neglected.
- For the given categories, the available official apps were selected based on their popularity, i.e., more than 1M+ downloads in the Google Play Store. Note that for certain devices, there may exist more than one official app. For instance, TP-Link offers at least three official apps, namely “TP-Link Tapo”, “Kasa Smart” and “TP-LINK tpCamera”, which can be used to manage an IoT device.
- Apps that have been analyzed in the past in the context of other research, including that in [1] have been excluded. For instance, the “Kasa Smart” app mentioned in the previous bullet point falls into this category.

All the apps’ noteworthy details are summarized in Table 1 following an alphabetical order, first per IoT group and then per app name in that group. The same sorting order is used for the rest of the tables and figures across the rest of the sections of this work.

Most of the included apps are applicable to multiple devices, i.e., operate across all devices that belong to a specific IoT category. Interestingly, the majority of the apps, including *Samsung SmartThings* and *MyFRITZ!App*, can manage multiple devices across different IoT categories. Therefore, for instance, the *MyFRITZ!App* is included in the smart assistant IoT category, as being the more relevant one. Based on November 2021 data [2], the distribution of Android users across the different versions of this operating system (OS) is, 3.7%, 5.1%, 11.5%, 14.2%, 28.5%, and 33.3% for v6, v7, v8, v9, v10, and v11, respectively. For this reason, Android versions prior to v6 are excluded from this study.

**Figure 1:**
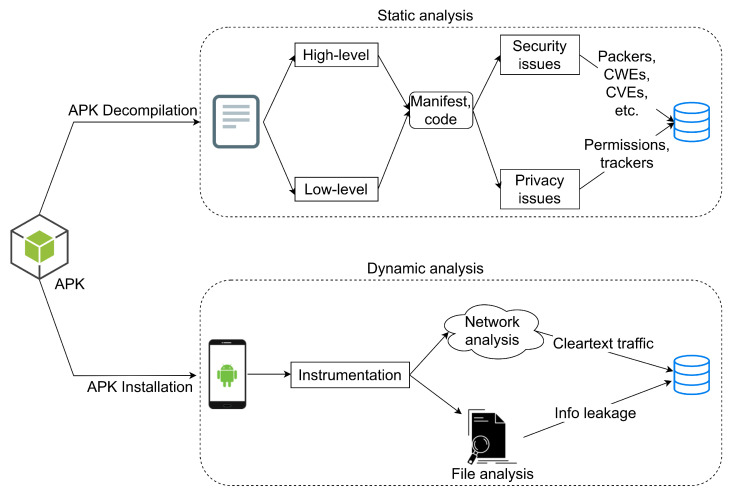
Overview of the followed methodology.

As illustrated in Figure 1, two basic axes of analysis were pursued. First, each app was examined statically. This type of analysis comprises several steps, including (a) sensitive permissions, and third-party trackers, which target mainly the privacy of the end-user, and (b) misconfigurations, weaknesses and vulnerabilities, which refer to the security level of the app. In a second phase, the most interesting apps in terms of potential vulnerabilities and popularity, were scrutinized dynamically, i.e., by running them, and when possible, pairing the app with a real IoT device. This phase concentrates on network traffic and file analysis, that is, the files generated by the app may contain sensitive information. For examining any possible functionality, each app was exercised by hand, that is, not through the use of a UI/app exerciser. For both the analysis phases, when searching for weaknesses and vulnerabilities, we followed the methodology set out in the OWASP mobile security testing guide [3]. Actually, more or less the same methodology have been followed by [4] regarding static analysis, [5,6] regarding dynamic analysis, and [7,8] for both types of analysis.

## 3. High-Level Static Analysis

Android permissions are split into three types, namely, install-time (it includes the normal and signature subtypes), runtime, and special. The focus of this section is on the penultimate type, which was introduced in Android 6 and evolved further in Android 10. A runtime permission is used to provide access to sensitive data the app may request, say, the user’s current location is safeguarded by a runtime permission. Given that a runtime permission has a potential to misuse, it requires a dialog prompt. That is, the user has to explicitly agree to grant such a permission or not; Android 10 provides increased transparency enabling the user to always allow, allow while in use, or deny a “dangerous” permission (The Android API marks such permissions as “Protection level: dangerous”).

Moreover, starting from Android 10, a runtime permission can be either hard-restricted or soft-restricted. Such a restriction is annotated in the *AndroidManifest.xml* as *hardRestricted* or *softRestricted*. The app installer, say, the Google Play Store may choose to not whitelist the restricted permissions for an app which does not conform to the platform’s policy. If whitelisted, the permissions behave normally. If not, the behavior depends on whether the permission is hard- or soft-restricted. A hard restriction means that the app cannot be granted a permission which is not whitelisted. On the other hand, a non-whitelisted soft restricted permission will behave as defined in the *SoftRestrictedPermissionPolicy*, that is, the public documentation for the requested permission. Note that a user cannot manually whitelist a permission.

### 3.1. Permissions

The identification and study of the runtime permissions used by an app is the initial step towards understanding its behavior from a privacy viewpoint. To this end, this subsection summarizes our findings regarding the runtime permissions identified in the *AndroidManifest.xml* file of each app included in Table 1. For easing the understanding of the detected permissions, we group them into six custom categories, namely *Utility*, *Authentication*, *Location*, *Storage*, *Phone*, and *Communication*. Specifically, the grouping of the various permissions in the different categories has been done with three goals in mind: (a) easing the reading of the current section, (b) improving the presentation of the results included in Table 2, and more importantly, (c) yielding useful discussions or conclusions based on the permission category rather than on each individual permission. It is implied that every dangerous permission identified in the examined apps has been included in the best matching category.

#### 3.1.1. Utility

This category includes every dangerous permission an app requested and is related to a sensor or a hardware component of the mobile device.

- U1: CAMERA requests direct access to the camera.
- U2: RECORD_AUDIO allows an app to record audio.
- U3: BODY_SENSORS requests access to different sensors that are responsible for measuring the user’s heart rate, steps, etc.
- U4: ACTIVITY_RECOGNITION allows an app to recognize when a user performs an activity, including heart health statistics, calories burned, training status, and others.

#### 3.1.2. Authentication

It is related to every dangerous permission that pertains to authentication methods and user accounts in general.

- A1: USE_CREDENTIALS allows an app to gain access to authentication tokens. It has been deprecated since API level 23 (v6).
- A2: AUTHENTICATE_ACCOUNTS allows an app to handle the account authenticator, i.e., a part of the AccountManager. The app can also create accounts and get/set their passwords. It has been deprecated since API level 23 (v6).
- A3: GET_ACCOUNTS allows an app to gain access to the accounts that are in the Account Service.
- A4: MANAGE_ACCOUNTS allows an app to manage accounts, including, creating and deleting accounts. It has been deprecated since API level 22 (v5.1).

#### 3.1.3. Location

The location category relates to location-based dangerous permissions.

- L1: ACCESS_FINE_LOCATION allows an app to learn the precise location of the user.
- L2: ACCESS_COARSE_LOCATION allows an app to access the approximate location of the user.
- L3: The hard-restricted ACCESS_BACKGROUND_LOCATION allows an app to access the location of the user, when the app is running in the background. Based on the Android documentation, the app must also request either one of L1 or L2 permissions to eventually gain access to the user’s location.
- L4: ACCESS_MEDIA_LOCATION introduced in API level 29 (v10), allows an app to obtain access to any shared geographic location existing in the user’s shared collection.

#### 3.1.4. Storage

This category comprises dangerous permissions which allow an app to access the OS filesystem.

- S1: READ_EXTERNAL_STORAGE grants read access to the external storage of the device, such as an SD card.
- S2: WRITE_EXTERNAL_STORAGE allows an app to gain write access to the external storage of the device.
- S3: REQUEST_INSTALL_PACKAGES allows an app to request and possibly install packages. According to the Android API, this is a signature type permission. Nevertheless, it is often flagged as a dangerous one, because it permits the app to install packages outside the Google Play Store.
- S4: MOUNT_UNMOUNT_FILESYSTEMS permits an app to mount or unmount files for removable storage. This permission requires platform level privilege, and therefore cannot be used by third-party apps. Previous work [9] demonstrates that this permission can be utilized for malicious purposes.

#### 3.1.5. Phone

This category includes dangerous permissions that have to do with phone management, such as reading or writing contacts and reading phone logs.

- P1: READ_PHONE_STATE was added with API level 26 (v8). It allows an app to gain read only access to the phone state, including the cellular network, any active calls the user may have, and to the list of any PhoneAccounts object registered on the device.
- P2: SYSTEM_ALERT_WINDOW permits an app to create windows on top of other already running apps. The Android API states that “very few apps should use this permission; these windows are intended for system-level interaction with the user” and “If the app targets API level 23 or higher, the app user must explicitly grant this permission to the app through a permission management screen.” Previous works [9,10] have shown that this permission is exploited by major malware families.
- P3: READ_CONTACTS allows an app to gain read only access to the user’s contacts.
- P4: WRITE_CONTACTS permits an app to gain write access to the user’s contacts.
- P5: READ_PHONE_NUMBERS is a subset of the P2 permission, and it was introduced with API level 26 (v8.0). This permission authorizes an app to obtain read access to the device’s phone numbers.
- P6: WRITE_SETTINGS is flagged as dangerous starting from API level 23 (v6). By granting this permission, the app gains read/write access to the system settings of the device.
- P7: GET_TASKS has been deprecated since API level 21 (v5). For the sake of backwards compatibility, this permission will still return some data, such as the app own data. As a result, an app can be allowed to retrieve information about currently and recently relevant running tasks. As with S4 and P3 permissions, according to [9], the current permission has been used by three different malware families.
- P8: READ_LOGS allows an app to gain read access to low-level system log files. As with S4, the current permission is not for use by third-party apps. Interestingly, the related work [9] have shown that this permission is frequently abused different malware families.
- P9: READ_CALENDAR allows an app to gain read access to the user’s calendar data.
- P10: WRITE_CALENDAR grants an app write access to the user’s calendar data.

#### 3.1.6. Communication

This last category contains dangerous permissions that are needed by an app to communicate externally. This includes among others SMS and call-based permissions.

- C1: RECEIVE_SMS is a hard-restricted permission. It allows an app to receive SMS.
- C2: READ_SMS permits an app to read SMS. It is hard-restricted.
- C3: SEND_SMS authorizes an app to send SMS, and it is hard-restricted as well.
- C4: RECEIVE_MMS is a hard-restricted permission. It allows an app to receive MMS.
- C5: READ_CALL_LOG grants an app read access to the user’s call log. It is hard-restricted.
- C6: CALL_PHONE allows an app to place a phone call without user confirmation.
- C7: USE_SIP permits an app to use the Session Initiation Protocol (SIP) service.
- C8: ANSWER_PHONE_CALLS was added in API level 26 (v8). It allows an app to answer phone calls.
- C9: PROCESS_OUTGOING_CALLS has been deprecated since API level 29 (v10). This is a hard-restricted permission, which authorizes an app to learn the number being dialed in the context of an outgoing call. This means that the app can redirect the call to a different number or abort the call completely.

### 3.2. Discussion

Table 2 gathers and groups the above said permissions as detected in the *AndroidManifest.xml* file of each examined app. As observed, across the six categories of permissions, a total of 35 different permissions were identified. From an IoT app category perspective, most apps across each IoT category were found to request U1 and U2 permissions granting access to the smartphone camera and record audio. Although the U2 permission seems natural for the Wireless IP cameras and Smart assistant app categories due to the nature of their utilization, as a rule of thumb, both U1 and U2 should not be applicable without relevant reasoning to the AP, Smart TV, and Smart bulb/plug categories. The U3 body sensors’ permission was detected in one app only. As it concerns the U4 activity recognition permission, as expected, 8 out of 12 Smart wearable apps were found to request it. Questionably, the same permission was detected in one app from the Smart assistant category.

With reference to permissions that fall under the authentication category, no more than 17% of the first 5 IoT categories were identified to request A1 and A2 permissions. Generally, A1, A2, and A4, which are all deprecated, are used by 8, 7, and 8 apps, respectively, which naturally is not a positive result. On the plus side, no more than 19% of the examined apps requested access to the phone manager, i.e., the A4 authentication permission, regarding the creation and deletion of accounts.

With respect to location-based permissions, surprisingly, more than 95% and 78% of the apps were found to gain access to L1 and L2 precise and coarse location permissions, respectively. Although both these permissions may seem natural for the smart wearable category, from a bird’s eye view, they are only marginal or totally unnecessary for the rest of the categories. Lastly, only one app was found to request access to the user’s shared location(s).

With regard to the storage category, the most populous permissions are S1 and S2. Specifically, more than 92% of the apps spread across all categories were identified to request read and write access to the external storage of the device. On the other hand, overall, a dozen of the apps in all but one category potentially permit the installation of packages outside the Google Play Store (S3). Naturally, this outcome is translated negatively. Moreover, only 4 apps included in the Smart TV, Wireless IP cameras and smart wearable IoT categories were found to allow mounting and unmounting of the device’s removable storage.

The most populous permissions in the Phone category are P1, P3, and P7; recall that the latter is deprecated since v5. P2 and P6 were also detected in a significant mass of apps, which seems at the most unnecessary or unjustifiable. The P1 to P3 permissions are included in the great majority of the apps in the Wireless IP cameras and Smart wearable categories, while for the latter category this may seem logical, there is not a particular reason for including such permissions in the former. Even more, it remains highly questionable why a Smart bulb/plug app might request and gain permission to phone type of permissions. No less important, according to the documentation provided in Section 3.1, P8 is unjustifiably present in a quartet of apps. Finally, communication permissions are mostly present in the Smart wearable category, and partially in the Smart assistant one, which is generally sensible. Strangely, however, some of this type of permissions were identified in the Wireless IP cameras as well.

Concluding the above discussion, as a general principle, the number of runtime permissions an app requests must be reduced to the bare minimum. Simply put, by restricting access to dangerous permissions reduces the risk of inadvertently misusing any of them, and in addition substantially aids in decreasing the app’s attack surface. Therefore, although a certain permission may appear essential or alluring for supporting a functionality, it may eventually undermine user’s adoption and augment the app’s susceptibility to assaults.

## 4. Low-Level Static Analysis

To further investigate each IoT app, and for Section 4.1–Section 4.5, we took advantage of the well-known Mobile Security Framework (MobSF) in v3.4.3 [11]. MobSF is one of the all-in-one tools suggested by the OWASP Mobile Security Testing Guide [3].

As shown in Table 3, low-level static analysis concentrates on a number of issues, including *Janus*, network security, signer certificate information, weak cryptographic hash functions used to sign the Android application package (APK), code analysis aiming at divulging Common Weakness Enumerations (CWE), tracker analysis, manifest analysis, and shared library binary analysis. For all the aforesaid categories, and for reasons of brevity, we point out only high value (severity) weaknesses according to the common weakness scoring system (CWSS). Therefore, low to medium value weaknesses are intentionally left out, except stated otherwise. For extracting these pieces of information, MobSF decompiles the APK using Dex to Java decompiler *(Jadx)* [12]; code de-obfuscation processes may apply to this step as well.

The last two subsections of the current section concentrate on the use of outdated third-party software by the apps and on taint analysis. For both these types of analysis, the *Ostorlab* tool was employed. This is a renowned software-as-a-service (SaaS) product used to review the security and privacy of mobile apps. For ensuring the reproducibility of the results, we used the free-to-use “community” edition of the tool. It is to be noted that Ostorlab has been utilized in the context of analogous vulnerability analysis researches, including the ones in [7,8,13,14].

### 4.1. Network Security and Certificates

A vulnerability, which roots in improper signature usage, is well-known as *Janus* (CVE-2017-13156). Janus can be exploited in cases where the v1 signature scheme (JAR signing) is used along with Android API 21 (v5) to API 25 (v7). Precisely, it leverages the possibility of adding extra bytes to APK and DEX files, without affecting the signature. As shown in Table 3, 36 of the 41 (or ≈88%) examined apps were found vulnerable to *Janus*, namely they were signed under scheme v1 and are compatible with Android v6.

On the other hand, as shown in the third column of Table 3, network security analysis revealed a number of high severity vulnerabilities. The first and more severe pertains to cleartext communications, i.e., the app is misconfigured to possibly allow unencrypted outbound traffic towards any network domain. The second, may allow cleartext traffic, but only towards certain domains. The third, denotes that the app trusts the system’s certificates; note that from Android v8, a system certificate can be added only if the device is rooted, hence this issue primarily affects versions prior to v8. Another issue is that the app trusts user installed certificates. This is because a non-security-savvy user may be lured into installing a malicious certificate. The last issue arises when the app is allowed to bypass certificate pinning; this may facilitate man in the middle (MitM) attacks. It is to be noted that all the aforesaid misconfigurations refer to the network security configuration XML file an app may designate through a special entry <application android:networkSecurityConfig=“”> in its manifest file under the <application> tag [15]. According to our analysis, 16, 2, 7, 3, and 1 apps were found to be vulnerable to the above-mentioned network issues, respectively.

Each APK is signed by the developer using a cryptographic hash function, e.g., SHA-1, and APK signature scheme version, e.g., v3. If the app has been signed using SHA-1 (or MD5), collisions may exist. In simple terms, apps signed with deprecated algorithms are prone to attacks, including hijacking the app with fake updates or granting permissions to a malicious app. For instance, the assailant may be able to repackage the app after embedding malicious code in it. Then, given that the signature validates, they could phish users to install the repacked app instead of the legitimate one. As seen from the fourth column of Table 3, a total of 27 apps use a deprecated cryptographic hash function to sign the app. From them, 21 declare the use of SHA256withRSA, but they actually used SHA1withRSA; the rest declare and indeed use SHA1withRSA. Recall that NIST deprecated the use of SHA-1 and suppressed its use for digital signatures in 2011 and 2013, respectively, [16].

The Android app identifier (APKiD) provides information about the way an APK was built. Precisely, APKiD is used by numerous static analysis tools to identify packers, i.e., agents created by packing engines used to protect the software. From them, the most pertinent one is *Packers* [17], originally produced to safeguard the intellectual property of apps. Nowadays, Android packers, including *Baidu* and *Bangle*, are used extensively by malware coders given that reverse engineering tools are typically incapable of unpacking and inspecting hidden payloads within packed apps. As seen from the fifth column of Table 3, four apps were found to utilize packing mechanisms.

#### Discussion

From an IoT category perspective, Table 3 reveals that all the apps in the AP, Smart Assistant, and Smart bulb/plug categories are susceptible to *Janus*. On the other hand, this vulnerability is present in half, 2 out of 9 (22%), and in 2 out of 12 (17%) apps, in Smart TV, Wireless IP cameras, and Smart wearable IoT categories, respectively.

As shown in the third column of Table 3, a significant number of apps in each IoT category were found to be prone to severe network security issues. Specifically, both the Smart TV apps, 3 out of 5 (60%) of the Smart assistant apps, and half of the Smart bulb/plug apps were identified to allow cleartext traffic towards any network domain. This figure is significantly lower in the remaining categories, with one-third, and 1 out of 12 or 8.5% apps, in the AP and Smart wearable categories, respectively. Moreover, 4 and 1 apps in the Smart wearable and Smart bulb/plug categories, respectively, were found to allow cleartext traffic towards certain network domains. On top of that, 3 or 33% and 25% of the apps in the Wireless IP cameras and Smart wearable categories trust the system’s certificates, respectively; this figure is also significant in the Smart assistant and Smart bulb/plug categories and around 11% in the AP one. On the plus side, the majority of apps do not trust user’s installed certificates. This situation is met only in the Wireless IP cameras and Smart wearable categories, but for a limited number of apps, namely 1 and 2, respectively.

The use of obsolete hashing schemes in APK signing is prevalent in all app categories except the bottom two in Table 3. Precisely, more than 55% of the apps in AP, Smart TV, Wireless IP cameras, and Smart wearable categories have been signed with SHA-1 algorithm, thus being prone to collision issues. Interestingly, this is the case with all but one app in the Smart wearable category. *Packers* is the most infrequently met issue across all the categories. Generally, only a couple of the IoT categories were found to incorporate a packing scheme.

### 4.2. Detected CWEs

The current subsection briefly discusses all high severity CWEs that are pertinent to each app. From them, CWEs 89, 276 and 502 belong to the list of top 25 most dangerous software weaknesses [18], while CWEs 295 and 532 occupy positions 28 and 33 in the extended list, respectively.

- *CWE-89*: This perilous weakness, titled “Improper neutralization of special elements used in an SQL command (’SQL Injection’)” is classified as M7 in OWASP-10. It is present in cases where the app does not sanitize or improperly sanitizes input stemming from an upstream component, e.g., from a Web form for user authentication. All but two apps (95%) were found to be potentially vulnerable to this issue.
- *CWE-250*: Known as “Execution with Unnecessary Privileges”, this weakness refers to any unnecessary elevated privilege the software may hold when performing an operation. Only one app were found to be susceptible to the relevant weakness.
- *CWE-276*: The so-called “incorrect default permissions” weakness, appears if the app is granted unneeded read/write permissions. In such a case, any affected file can be potentially read or written by third parties. By referring to OWASP-10, this weakness is classified under M2, namely, “insecure data storage”. All apps, but one, were found to be susceptible to this weakness for at least one of the below reasons. The first, is related to the creation of a temporary file, which may contain sensitive data. This is a significant issue, since anyone is able to access folders that contain temp files. The second, represented by an additional plus sign, relates to the fact that the app requests read or write access to the external storage.
- *CWE-295*: It is titled “Improper certificate validation” and classified under M3 in OWASP-10. This situation occurs if the app is configured to trust an insecure or self-signed or any sort of certificate during a Transport Layer Security (TLS) handshake. As already pointed out, this weakness may enable an attacker to mount MitM attacks. About 50% of the examined apps were found to be susceptible to this weakness. An additional plus sign in the same column designates that this app is susceptible to the same weakness for their *WebView* implementation as well.
- *CWE-312*: It is known as “Cleartext storage of sensitive information”, and is classified as M9 in OWASP-10. When sensitive pieces of information, say, a username and/or password, are kept in cleartext form, anyone can read them. In some cases, such sensitive information may be statically placed in the code of the app, e.g., in a configuration file. As seen from Table 3, the totality of the apps were found to be susceptible to the current weakness.
- *CWE-327*: The “Use of a broken or risky cryptographic algorithm” is classified as M5 (“Insufficient Cryptography”) in OWASP-10. This weakness pertains to the usage of outdated hash or encryption algorithms. It is observed that all the examined apps, but one may potentially use at least one obsolete hash algorithm, namely either MD5, SHA-1, or both, and 24 of them—those having a value of “3”—support AES-ECB, which is not semantically secure.
- *CWE-330*: This weakness titled “Use of insufficiently random values” relates to the generation of predictable random values by an app. It typically means that the app employs an insecure random number generator. In the OWASP top 10 mobile risks list, this weakness ranks in the fifth position, namely “insufficient cryptography”. All apps were found to be susceptible to this weakness.
- *CWE-502*: The app can possibly use an untrustworthy way of deserializing data, which is known as a “Deserialization of Untrusted Data” weakness. In our case, one app were found to use a *Jackson* deserialization library, which could possibly deserialize data in an unsafe way. Favorably, only one app presents this weakness.
- *CWE-532*: The weakness, known as “Insertion of sensitive information into log file”, arises when a production app has enabled logging information to a file, while this feature may be of particular aid during the development stage of an app, it must be removed prior to the app is made publicly available. If not, an attacker could read these files and obtain any private information stored in them. All apps were found to be susceptible to this weakness.
- *CWE-649*: The “Reliance on Obfuscation or Encryption of Security-Relevant Inputs without Integrity Checking” weakness refers to the usage of cryptographic algorithms that require the validation of the encrypted data. Simply put, an app that uses such an algorithm must perform integrity checks to validate that these data are not tampered with. More than thirty of the examined apps rely on AES-CBC with PKCS5/PKCS7 padding, without validating their encrypted results. This means that these apps may be vulnerable to the so-called padding oracle attacks.
- *CWE-749*: The “Exposed dangerous method or function” weakness belongs to M1 (“Improper Platform Usage”) of OWASP-10. It can arm several major vulnerabilities depending on the case at hand, that is, the underlying vulnerable function. In our case, 29 of the examined apps were found to offer an insecure *WebView* implementation. The latter is used to display Web content as part of an activity layout. In the presence of this weakness, an attacker could possibly perform a MitM assault or even execute a Cross Site Scripting (XSS) injection. For further information about this issue, we refer the concerned reader to the “WebView” section of [19].
- *CWE-919*: The overarching “Weaknesses in Mobile Applications” view is related to CWE-749. In this case, both of them refer to the same issue, but for a different matter. Namely, we observed that 17 apps have enabled the remote WebView debugging. It is common knowledge that this particular mode must be disabled before deploying a production app. If not, anyone who can access an unlocked mobile device can easily acquire access to the app’s data.

#### Discussion

All the app categories were found to be susceptible to several CWEs, as presented in Table 3. Even more, all apps across every IoT category were found to be susceptible to CWEs 312, 330, and 532. Almost the same picture applies to CWEs 276 and 327; all apps but one are found to be susceptible to these weaknesses. The situation is more or less better for CWEs 649, 749, and 295, demonstrating a susceptibility percentage of around 75%, 66% and 46%, respectively. On the positive side, both CWEs 250 and 502 have an insignificant presence among the apps, possibly affecting only one app in both the Wireless IP cameras and Smart assistant categories.

### 4.3. Tracker Analysis

This subsection centers on third-party trackers that may be present in each app. As already pointed out, for this task, we relied on MobSF, which uses the open source *Exodus-Privacy* [20] Web app to characterize in a coarse manner any tracker found in the app’s source code. We concentrate on 7 categories of trackers. The first is “Crash reporters”, which relates to the crashes that may occur during the normal operation of an app. That is, following an app crash event, they send a notification message to the developers, informing them about the error. The next category is known as “Analytics”. Such a tracker collects any information regarding the usage of the app by the end-users, say, the time each user spent in the app, which features they used, and so forth. Next, are “Identification” trackers, which basically attempt to identify the end-user.

A “Customer support” tracker on the other hand provides the app with a *chatbot*, capable of assisting the users in real time; while this category may seem quite irrelevant, it should be pinpointed that the messages a user has exchanged with the chatbot is most probably in the possession of the tracker too. An “ads” tracker is specialized in serving personalized advertisements to the users. “Location” trackers gather data that stem from location services and the different sensors the mobile device may incorporate. For instance, after the tracker learns the geographical location of the user, they can be targeted with location-based ads. Lastly, a “Marketing” tracker, applies a set of personalization, identification, and location-based techniques to assist the app developers to better analyze the app’s users, i.e., profile them. Usually, this generic category embraces trackers that belong to at least two tracker categories. For instance, the *Braze* tracker is categorized as both analytics, ads, and location. For this reason, any such tracker is included in the marketing category.

For more details regarding the third-party trackers’ phenomenon in the mobile ecosystem, the interested reader can refer to the works by [21,22,23].

Our analysis showed that only two out of the total 41 apps contain zero trackers (4.87%). On the downside, a couple of apps utilize more than 10 trackers each. Overall, 39 unique trackers were identified across all apps. Among them, Google *Firebase* and Google *Analytics* are included in 32 (78%) and 5 (12.2%) apps, respectively. Additionally, all apps but two have at least one Analytics tracker. Another commonly met tracker is the Google *CrashLytics* one; 24 apps or 58.5% contain this tracker. Moreover, 11 or 26.8% of the examined apps incorporate at least one Facebook tracker, namely, *Login*, *Analytics*, *Places*, and *Share*, detected in 11, 6, 6, and 10 apps, respectively. Interestingly, 5 out of the 39 apps contain an Ads type of tracker, 11 of them embed Identification trackers, and only a couple of apps include a Customer support tracker.

**Figure 2:**
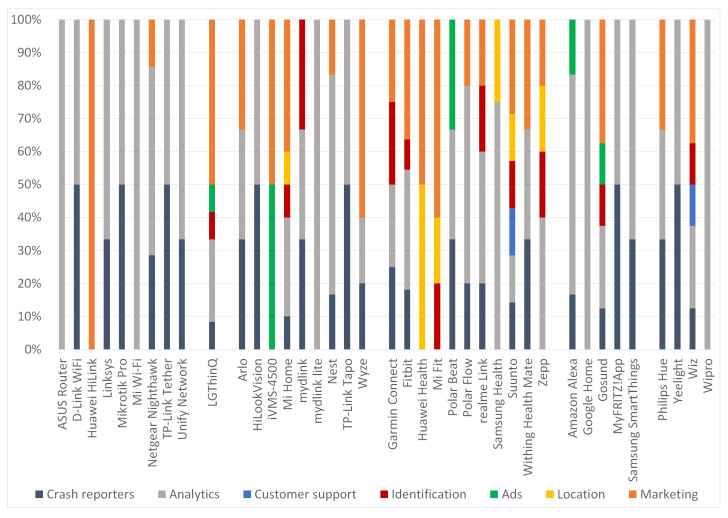
Categorization of trackers per examined app.

With reference to Figure 2, the AP category has mostly Analytics or Crash reporter trackers. Moreover, 2 out of the 9 apps in this category were found to have at least one Marketing tracker. On the other hand, the single app in the Smart TV category demonstrated an assortment of trackers, with the Marketing category to be the most populous. Analytics and Marketing trackers are common in the wireless IP cameras category, while Ads and Location type of trackers were rather scarce in the same category of apps. All apps, but two in the Smart wearable IoT category, contain Marketing trackers, most of them Analytics, and several of them Location ones. The Analytics type of trackers is also the most frequently met in the Smart assistant and Smart bulb/plug categories. Overall, the most frequently met categories of trackers across all the apps were the Analytics, Marketing, and Crash analytics (in that order).

From a total of 39 unique identified trackers, the Analytics and the Marketing categories include the 27 (15 and 12) of the trackers, while the Crash reporters follow with 5 trackers. Every detected tracker is given in the following list.

- *Crash analytics*: Bugsnag, Bugly, Google CrashLytics, Instabug, Microsoft Visual Studio App Center Crashes.
- *Analytics*: Amazon Analytics, Dynatrace, Google Firebase, Google Analytics, Google Tag Manager, Keen, Metrics, Facebook Analytics, Microsoft Visual Studio App Center Analytics, New Relic, Optimizely, OpenTelemetry, Sensors Analytics, Splunk MINT, Tencent Stats.
- *Identification*: Facebook Login.
- *Customer support*: HelpShift.
- *Ads*: Amazon Ads, Flurry, Google AdMob.
- *Location*: AutoNavi, Mapbox.
- *Marketing*: Adobe Experience Cloud, Braze, Display, Facebook Places, Facebook Share, Huawei Mobile Services (HMS) Core, MixPanel, Salesforce Marketing Cloud, Segment, Swrve, Treasure Data, Urbanairship.

#### Discussion

**Figure 3:**
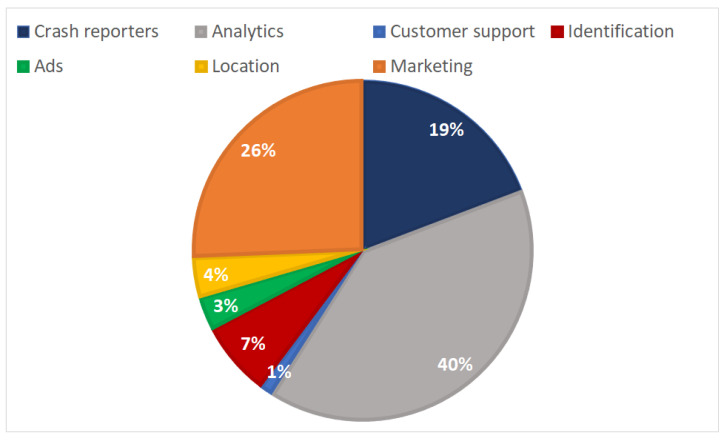
Allocation of trackers per category.

Overall, a total of 156 trackers were detected across all the examined apps. As observed from Figure 3, the Analytics and Marketing categories accumulate the majority of trackers; 62 or 40% and 40 or 26% trackers fall into these two categories, respectively. It is also perceived that the Ads, Identification, Location, and Marketing categories, which are considered more *privacy-invasive* from the rest, sum up to 40% or 62 trackers.

From an IoT category perspective, two apps out of 10 in the AP set incorporate one Marketing tracker. The greater number of *privacy-invasive* trackers were detected in one app belonging to the Smart TV category. Regarding the Smart wearable and wireless IP cameras categories, 5 out of 12 apps (41%) and 9 out of 11 (82%) have at least one Marketing tracker, respectively. On top of this, the Smart wearable category presents the higher number of Identification and Location trackers. Precisely, 50% of apps in this category have at least one Identification or Location tracker. The privacy concerns here are graver because such apps are widely used to manage fitness trackers. On the positive side, the remaining two categories, namely Smart assistant and bulb/plug, present only one and two apps with a marketing tracker, respectively. Appendix A.1 names all the identified trackers per app.

### 4.4. Manifest Analysis

Based on the results of the static analysis given in Section 3, we pinpointed the use of several potentially privacy-invasive permissions, with several of them not really necessary for the proper functioning of the app. In a further step, this section delves deeper in the manifest file of each app to possibly reveal any hidden weaknesses.

#### 4.4.1. Services, Activities, and Broadcast Receivers

This subsection concentrates on services, activities, and broadcast receivers, which are materialized through intents and intent-filters. Based on the Android developer’s guide, this triad of components should be declared in the manifest file of the app [24].

*Intents* comprise message objects for serving intra- or inter-app communication needs. Specifically, such objects can be used to launch an activity, start a service, or convey a broadcast. An intent can be either explicit or implicit. The first type is used for relevant messages within the app, while the second to deliver messages towards another app. An *intent filter* is an expression in the app’s manifest file that specifies the type of intents the specific component would like to receive. This means that an intent-filter takes care of implicit intents, including broadcast receivers, and capturing of system-oriented broadcast messages, where the corresponding broadcast message exists in an intent. Simply put, a matching intent-filter must be present in the manifest file of an app for the latter to receive such a broadcast message. In a few cases, such as the D-Link WiFi, the app implements a high intent priority, potentially overwriting any other intent-based request. This implementation can potentially enable a malicious actor to extract protected information, such as the passphrases of the Wi-Fi network.

A *service* app component is able to execute operations without the aid of a User Interface (UI). A typical usage of such a component is to transparently transfer files from an app to another. Three types of services exist: *foreground*, which is visible to the user, *background*, which is not directly visible, and *bound*. The latter is used for binding a certain service with an app. An *Activity* on the other hand is an essential component of Android apps. Basically, it corresponds to an individual, specific action the end-user can carry out, and therefore it usually requires a UI. For example, opening an app normally involves the execution of the so-called *main activity*.

In the course of app analysis, it was perceived that numerous of such components neglect to declare the corresponding permission in the app’s manifest file. That is, based on [19], for enabling a component to communicate with other apps, the developer must declare it in the manifest file. However, for curbing this functionality, the developer must also declare a matching permission. This means that only apps that also include the same permission will be able to communicate with this component. Naturally, this is a baseline protection and it is not adequate to bulletproof the component. In any case, skipping to include the correct permission to the component is reckoned as a weakness of high severity.

The relevant results gathered for each app are summarized in Table 4. The left half of the table splits the components in two classes, that is, intent-filter on and off. The former designates that the app is able to dispatch and accept intents which target the relevant component. If the incoming intent is malicious, it may compromise the app, as for example explained in [25]. The latter (off) designates that the app is only allowed to transmit data upon receiving a request from another app. In this respect, an app may unknowingly divulge sensitive information to an attacker. By observing Table 4, it is concluded that in either of these two categories and across all components types, a significant portion of the apps, i.e., 22, 28, 33 and 22, 22, 28, respectively, omit at least one time to set out the right permissions.

#### 4.4.2. Other Issues

A content provider [26] is an app component destined to interplay with a repository. Such a component are useful for “supplier” apps, i.e., those that provide data to others. In this sense, any content provider should by default allow “consumer” apps to reach its data. However, in case the <provider> manifest element declares no permissions for a content provider, any other app can obtain read and write access to it. By referring to the first column of the right half of Table 4, 14 apps were found to be potentially vulnerable to such an issue, omitting to declare the correct permission(s) in its manifest file.

Moreover, from the *Task* column of the same table, it is observed that 11 apps have enabled at least one task affinity for a given activity, in which the intent-filter was turned off. Precisely, task affinity [27] relates to an activity that will be re-parented to, using the proper attribute (*allowTaskReparenting*). Furthermore, the task affinity is linked to the main activity; the latter will be launched upon calling the *FLAG_ACTIVITY_NEW_TASK*. Bear in mind that by default, every app has the same task affinity. This is a significant matter given that an evildoer may be able to eavesdrop on intents transferred among activities. For instance, we refer the interested reader to CVE-2020-0096, commonly known as *Standhogg 2.0*. Namely, this vulnerability may take advantage of the above-mentioned issue in unpatched Android v8, 8.1, and 9.

The five rightmost columns of Table 4 contain information about a handful of other important manifest file issues, namely, *Launch*, *Cleartext*, *Backup*, *Priority*, and *SMS*. The first is related to the Launch mode, that is, the *launchMode* attribute in the <activity> manifest element, which controls the way an activity will be initiated. As observed from the table, 14 apps declare a disparate Launch mode for their main activity, which typically is launched using the *default* mode. For instance, one app in the wireless IP cameras category has 72 activities which are configured to be launched differently. Precisely, every such app has set its launch mode to *singleTask/singleInstance* [28] (singleton). Note that the singleton design pattern restricts the instantiation of a class to only one instance. Nevertheless, under this mode, the app can control other activities (known as singleTop activities), which have the standard configuration. Furthermore, every activity having the *singleInstance* launch mode is always the root activity of a task, meaning that and no additional activities will be created for the same task. As stated before, this issue is also connected to the task affinity. The work in [29] details on how an attack can capitalize on this particular matter.

The *Cleartext* column refers to the *android:usesCleartextTraffic* flag in the <application> manifest element. It designates whether the app intends to use cleartext network traffic, including cleartext HTTP. As observed from Table 4, 12 apps permit this functionality. Obviously, this may jeopardize user’s privacy or even leak their credentials. This situation should be reckoned in conjunction with the “Network security” column of Table 3. That is, considering the unique instances in the respective columns of both tables, 25 apps in total allow cleartext traffic to any domain.

Android apps provide the option to be backed up via Android debug bridge (adb); the user needs to enable the USB debugging functionality. Naturally, for exploiting this functionality, the attacker needs physical access to the device. With reference to the *Backup* column of Table 4, 6 apps were found to have this functionality enabled.

The next to last column of the table, namely *Priority*, has to do with the intent priority the app defines. If this priority is set, the current intent can override other requests and potentially cause unexpected results. Eight apps were found to have assigned high priority to specific intents.

The *SMS* column refers to a situation where an app has been configured manually to accept binary SMS, and has bound this service with a specific port, in our case the 8080 one. If the app does not properly validate this service delivered in binary format, an attacker could potentially craft and send malicious SMS to the app, which may lead to unexpected results. Only one app was found to be exposed to this issue.

#### 4.4.3. Discussion

With reference to the left half of Table 4, a plethora of apps across every IoT category were found omitting at least one time to declare the right permissions relevant to the three components of each intent-filter category (on or off). More than 50% of the apps across the first four IoT categories were identified to have a maximum of 4 services related to the intent-filter on component of transmitting and receiving all kind of intents. Nevertheless, one app in the Smart wearable category was found to exceed that number, with 22 different intent services. On the plus side, no more than 25% of the examined apps in the Smart assistant and Smart bulb/plug categories included intent-filter on services. The same percentages as with the aforementioned service component apply to its equivalent in the intent-filter off category. Precisely, more than 54% of the apps across all the categories were identified to have a range of 1 to 8 intent-filter off services and 2 of them surpassed that number with 13 and 10 services, respectively. Concerning the broadcast receiver component, more than 68% of the apps from all the IoT categories were identified to receive broadcast messages. The most populous IoT app category in both intent-filter columns (on and off) was determined to be the Smart TV category.

Regarding the end-user’s executed activities with intent-filter on, it seems rather peculiar that the vast majority (80%) of the apps across all the categories incorporate the ability to send and also receive intents targeting those components. At the other side of the spectrum, leakage of sensitive information due to the identified activities with intent-filter off is calculated to be around 68% of the total number of IoT apps.

With respect to the rest of the issues shown in the right part of Table 4, the most by far affected IoT category regarding content provider’s data handling is the Smart wearable one, demonstrating a percentage of 67%. No less important, the only IoT app category which handles content providers correctly is the Smart TV one. On top of that, task affinity activity components in which the intent-filter is disabled are also handled properly within the Smart TV apps. Regarding task components, more than 27% of the apps across the remaining categories have enabled a number of task affinities ranging from 1 to 9 per app. Nevertheless, one app in the Wireless IP cameras category was found to by far exceed that number, with 26 different task affinities enabled.

Regarding the Launch mode component, all but one of the IoT categories were found to incorporate apps with modified default Launch mode for their main activities’ execution. The most affected IoT categories regarding Launch activities are the AP and Smart bulb/plug ones. Five out of 9 (56%) and 75% of the apps belonging to the aforesaid categories, respectively, were identified as prone to this misconfiguration. This figure is substantially lower in the remaining three categories, with half of the Smart TV apps and no more than 16% of the Wireless IP cameras and Smart wearables apps being affected. Nevertheless, one app from the Wireless IP cameras, Smart wearable, and Smart bulb/plug categories exceeds by a great deal the rest of the apps in the number of different misconfigured launch components with 72, 49, and 35 detected instances, respectively.

Issues pertaining to cleartext traffic were mainly identified in the AP and Smart assistant IoT categories, with 5 and 3 apps, respectively. Only one app in each of the remaining categories were found to potentially allow for such a risky behavior.

At the bright side, the majority of the apps do not employ the backup functionality. This situation is scarcely encountered in all but one category of apps, and it is completely absent from the Wireless IP cameras one. Interestingly, the majority of the affinity priority components are identified within the apps of the Smart wearables category (41%), with no more than 22% of the aforementioned component being present to the AP and Wireless IP cameras categories, respectively. SMS is the least frequently met issue across all the categories; only one app in the Wireless IP cameras category was found to accept this type of messages.

### 4.5. Shared Library Analysis

This type of analysis aims at the shared libraries (with the extension *.so*) an app may use. Such libraries are usually written in *C* and compiled through the Android native development kit (NDK) tool set, and loaded into memory at runtime. Android capitalizes on this logic for delivering better performance and reusing existing C libraries, without porting them to Java. Security and privacy issues stemming from the use of shared libraries have been already analyzed in the Android literature [30,31,32].

**Figure 4:**
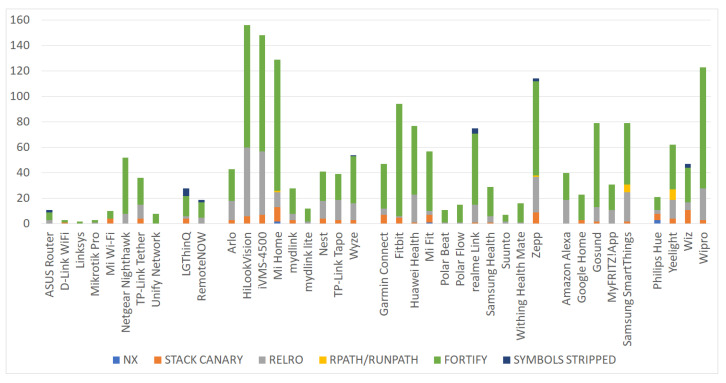
Number of identified shared library issues per app.

To explore if and to what degree this issue is applicable to the considered apps, we present the number of potentially vulnerable shared libraries per app in Figure 4. As observed from the figure, we identified six code hardening methods, namely no-execute (NX), Stack Canary, relocation read-only (RELRO), RPATH/RUNPATH, FORTIFY, and SYMBOLS STRIPPED, which if omitted may render the app vulnerable to serious vulnerabilities, such as buffer overflows or remote code execution exploits. More precisely, this type of countermeasures, known also for the first five of them as memory corruption mitigation techniques, are specific to C language, and if overlooked may create space for memory-based exploits, which inevitably migrate to the Android app. For instance, FORTIFY and SYMBOLS STRIPPED are linked to buffer overflow and reverse engineering threats, respectively, and therefore can potentially lead to arbitrary code execution from the one hand, or vastly improve the understanding of the underlying source code from an attacker’s viewpoint from the other.

An operating system that supports the *NX* bit can tag specific sections of the memory as non-executable. This way, the CPU will deny executing any code residing in that memory region. If the *NX* bit is not set on the library, an assailant may be in position to perform a buffer overflow. Namely, often, such attacks inject code in a program’s data region or stack, and next jump to it. If all writable addresses are non-executable (through the *-z noexecstack* compiler flag), such an attack is obstructed. As seen from Figure 4, a couple of apps were found to incorporate libraries that allow for executable writable addresses in memory.

*Stack Canaries* are a broadly-utilized defense against memory corruption assaults. A stack canary is a value placed on the memory stack with the purpose of being overwritten by a stack buffer that overflows to the return address. If this protection is absent, the app is vulnerable to legacy stack buffer overflow attacks. With reference to Figure 4, 26 apps embrace shared libraries, which disregard this countermeasure.

*Relocation Read-Only* (RELRO) is a mechanism to harden Executable and Linkable Format (ELF) binaries by declaring some binary sections read-only. Precisely, *RELRO* ensures that the Global Offset Table (GOT) lookup table used by a dynamically linked ELF binary to dynamically resolve functions that exist in shared libraries, cannot be overwritten [33]. As seen in Figure 4, 34 apps neglect this defense.

On the other hand, *RPATH/RUNPATH* is used to set the runtime search path for library search. This path is defined in the APK. Put simply, this path information is used by the linker to locate the shared libraries. An issue may arise if the attacker has also access to the same path. If so, then the attacker can inject arbitrary code to any shared library. To prevent such a situation, the flag *-rpath* must be removed during compilation. Less than 5 apps were found to be vulnerable to the relevant issue.

*FORTIFY* is a set of C standard library extensions that attempts to detect the erroneous usage of standard functions, including *memset*, *memcpy*, *sprintf*, *strcpy*, *gets*, and others at compile or run time. When compiling the app using the *D_FORTIFY_SOURCE = 2* flag, several buffer overflow errors related to various string and memory manipulation functions can be identified. Specifically, a detected buffer overflow at runtime terminates the program, while a detected bad call to a standard library function at compile-time, generates a compilation error. All apps but one, were found to have been compiled without using the *D_FORTIFY_SOURCE* macro.

The compiled code should be given with the minimum explanation possible. That is, certain metadata, including debugging information, and descriptive function or method names, render the binary or bytecode simpler for the reverse engineer to parse and comprehend. For instance, if an executable is compiled with gcc’s *-g* flag, it will include debugging information. For preventing this issue, symbols and other debug information must be stripped from the executable; we refer to this issue as *SYMBOLS STRIPPED*. This is done by compiling the executable using the *-s* or the *-g0* option. The first removes all symbol table and relocation information from the executable, while the second produces no debug information at all. Seven apps were found to not be using such options, and therefore are far more prone to reverse-engineering.

#### Discussion

**Figure 5:**
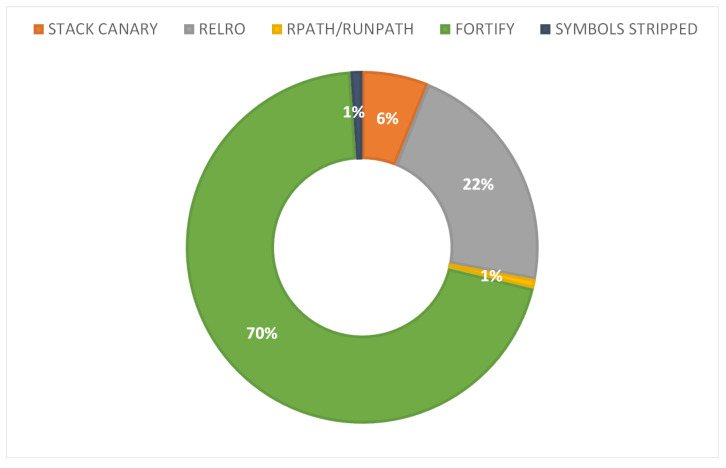
Breakdown of shared library issues. The NX value is insignificant, i.e., less than 0.5%, and it is not included in the figure.

A total of 1867 of shared library issues were identified across all the examined apps. Precisely, the Fortify C standard library extensions mitigation technique and *RELRO* ELF binaries hardening mechanism accumulate the great majority of the cases as they account for a total of 1307 and 407 issues across all apps, respectively. As illustrated in Figure 5, the aforesaid numbers are translated to 70% and 22% of the total number of issues. The great mass of the rest of the cases refer to *STACK CANARY* misconfigurations, which were found to be 113 or 6% of the total number of issues. The remainder of the identified issues pertain to *NX*, *RPATH/RUNPATH*, and *SYMBOLS STRIPPED*, with 6, 16, and 20 cases across all the examined apps, respectively.

From an IoT category perspective, the most affected IoT categories by the *FORTIFY* issue were the Wireless IP cameras and Smart wearable. The Wireless IP cameras, Smart wearable, and Smart bulb/plug categories were found to be the ones with the greater number of *STACK CANARY* issues. Precisely, 8, 12, 4 apps from the aforementioned categories were susceptible to 40, 30, and 23 *STACK CANARY* issues, respectively. Most *RELRO* issues were identified in the previous 3 IoT categories and in the Smart assistant one. Specifically, this kind of misconfiguration was identified in every app of these categories, but one app in Smart assistant, with 181, 83, 64, and 49 issues, respectively. Lastly, the *NX*, *RPATH/RUNPATH*, and *SYMBOLS STRIPPED* misconfigurations had a rather limited presence across all the 6 categories of apps.

### 4.6. Outdated Software Components Analysis

Third-party components, say, libraries, comprise one of the cornerstones of modern software development. Nevertheless, as already pointed out in Section 4.5, the benefit of reusing third-party code may be largely void, if that code is buggy or obsolete. This may silently increase the attack surface of the app by far and render end-users vulnerable to security and privacy threats rooted in such external software components. Actually, the importance of updatability of such Libraries on the Android platform has been repeatedly diagnosed in the literature [34,35]. In other words, it has been demonstrated that many Android apps do not modernize their third-party libraries, remaining susceptible to a range of Common Vulnerabilities and Exposures (CVE).

As noted above, to delve deeper into this issue under the viewpoint of the examined apps, we employed the commonly accepted *Ostorlab* tool. The results of this type of analysis per app are outlined in Table 5. It is perceived that nearly the two-thirds (23) of the apps make use of one at least outdated library. As already stated, such an inadequacy is tightly connected to at least one CVE, meaning that the respective app is susceptible to publicly disclosed security flaws. Given the plethora of the involved CVEs, in the Appendix A.2, we succinctly refer to such issues per shared library by just enumerating the relevant CVEs, along with their severity. The interested reader may additionally consult the respective CVE page (https://nvd.nist.gov/ (accessed on 10 October 2021)) For the sake of brevity, we also leave out references to mainstream libraries such as OpenSSL.

#### Discussion

As observed, from Table 5, nearly 56% of the apps make use of at least one outdated library. It should be pinpointed that around 34% of the apps demonstrate one or more issue with the OpenSSL library, while nearly 22% of them expose a SQLite issue. Even more, 6 apps make use of obsolete versions of both the aforementioned libraries. Overall, the great mass of the problems is with reference to these two key libraries, followed by the OpenCV, Python, and jQuery ones (in that order).

Focusing on each IoT category, the wireless IP cameras category gathers the greater number of issues, followed by the AP one. What seems important to be pointed out is that app vendors for the aforesaid two categories of apps were identified as the most susceptible to a range of CVEs due to reuse of outdated versions of the OpenSSL library. Library issues in the rest of the categories are rather scanty. Clearly, the less affected category is the Smart TV one, but this is to taken as an indication only, given that the number of apps in this category is limited.

### 4.7. Taint Analysis

Static taint analysis, namely, a form of information-flow analysis, has been also done with the aid of the *Ostorlab* tool. This type of scrutiny can divulge possible data leakages in the examined code. This refers to an assortment of user or other kind of input sanitization flaws, that may facilitate Intent injection, SQL injection, or Command injection. Taint analysis uses a script, which tags every private data of interest, known as the *source*. Next, by tracing each source throughout the code, the analyst may become aware of each piece of code that potentially has a leakage, the so-called *sink*.

Because of the large number of intent-based issues this analysis yielded, we grouped the relevant problems into 3 categories, namely Intent Spoofing, SQL Injection, and Command Injection. As already mentioned in Section 4.4, Intent spoofing is related to 3 key components of the Android OS, namely, broadcast receivers, services, and activities. By appropriately sanitizing these components in the manifest file, leads as a matter of course to fewer leaked intents. SQL and Command injections on the other hand are rooted in more specific and potentially perilous weaknesses that need to be deracinated in a more urgent way.

**Figure 6:**
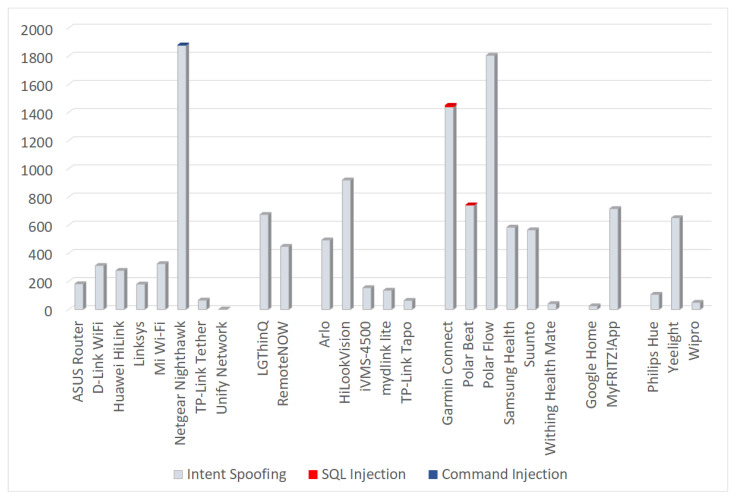
Issues identified through taint analysis. The Y axis designates the amount of issues per app.

Figure 6 provides a quantitative view of the results obtained after applying taint analysis to each of the considered apps. As illustrated, 26 apps were found to be susceptible to at least one issue, while three of them present more than 1400 potential issues each. There are also 9 apps that exhibit more than 400 (and less than 1000) issues each. Specifically, with reference to the Intent spoofing results, it was perceived that some apps leak usernames, email addresses, passwords, phone numbers, user sign-in credentials to Google Firebase or Spotify, access point characteristics (model, MAC address, firmware version, etc.), user authentication tokens, security questions for password recovery, camera data, user’s device characteristics, and details about user’s downloads. Last but not least, two apps in the Smart wearable category were found to be possibly vulnerable to SQL injection attacks, while one app in the AP IoT category was susceptible to Command injection attacks.

#### Discussion

From an IoT category perspective, as distinctively shown in Figure 6, the great mass of problems is with reference to Intent spoofing, as it was found to be applicable to more than 63% of the apps. Additionally, AP and Smart TV apps were the most affected to Intent spoofing, with 89% (or 8 apps) and 100% (or 2 apps), respectively. It should be noted, that the remainders of the identified issues pertaining to Intent spoofing represented no more than 50% of the examined apps per category. It should be pinpointed that SQL injection and Command injection potential vulnerabilities were scarce among the examined apps; only 5% (or 2 Smart assistant apps) and 3% (or 1 AP app) were found to be susceptible to these issues.

## 5. Dynamic Analysis

For the needs of dynamic analysis, we used a *Genymotion* rooted VM on top of an Oracle’s *VirtualBox* on Android v8.0. Currently, this is the latest version with an ARM translator (https://github.com/m9rco/Genymotion_ARM_Translation (accessed on 10 October 2021)). The latter is capable of installing Android apps without the need of Google Play Store; this avoids having all apps installed at the same time. After an app was analyzed, it was uninstalled, therefore, only a single app was installed in the VM each time. Moreover, MobSF was utilized as a dynamic instrumentation tool, which handled the dynamic analysis procedure. To achieve this, MobSF utilizes the well-known *Frida* server. It is to be noted that this section only concentrates on specific aspects of the apps, namely files containing sensitive information and network traffic. This means that we did not exhaustively exercise each app, say, through an app exerciser like *Monkey*, but we manually interacted only with the functionality an ordinary user will most probably utilize depending on the specific app type.

Another relevant issue has to do with Bluetooth connectivity, given that Genymotion (as with any similar VM) does not offer Bluetooth compatibility. Therefore, for the devices that required a Bluetooth pairing before being associated with the Android app, we paired the targeted IoT device with a user account, and then that account was used to obtain access to the device from the VM via a Wi-Fi connection. However, as shown in Table 1 some apps provide only Bluetooth connectivity. For these apps, we manually entered a set of representative measurements.

For connecting the VM to the Internet, the *adb shell settings put global http_proxy:0* command must be used (https://github.com/MobSF/Mobile-Security-Framework-MobSF/issues/1239 (accessed on 10 October 2021)). It should be noted that each IoT device was paired only with one app. Overall, the dynamic analysis is quite different per each IoT category based on two factors. First, by having already the results of static analysis, it was easier to discern the set of apps that presumably will yield a significant number of issues in the context of dynamic analysis. Second, from a consumer’s viewpoint, we cherry-picked the most popular apps along with a set of IoT devices, from each category. Overall, 13 different IoT devices were utilized: 6 APs, 3 wireless IP cameras, 3 Smart assistants, and 1 Smart bulb.

### 5.1. Access Points

For this category of devices, we tested 6 apps, namely, ASUS Router, D-Link WiFi, Linksys, Mi Wi-Fi, Netgear Nighthawk, and TP-Link Tether. The respective contemporary AP models were ASUS RT-AX88U, DIR-X1860, MR7350, AX1800, RAX40, and AX10v1, respectively. Excluding legacy or common Wi-Fi vulnerabilities [36,37,38], for such devices, an essential concern is the protection of the Wi-Fi passphrase, i.e., the key(s) to connect to the Wi-Fi network. Another major concern is the safeguarding of the user credentials used to connect to the AP’s web-based management interface. Under this prism, the dynamic analysis of these apps concentrated on searching for files where such credentials may be kept by the app in cleartext form.

- *ASUS Router*—This app uses the Link Layer Discovery Protocol (LLDP) to learn about and subsequently communicate with new APs. Following the first successful user login through the AP’s web-based management interface, the app keeps in its cache the administrator’s credentials. That is, the cleartext credentials are stored in the datadatacom.asus.aihomedatabasesrouterprofile file. It is noteworthy that this app uses an HTTP connection to communicate with the AP.
- *D-Link WiFi*—This app uses an HTTPS connection to communicate with the router. It was perceived that the passphrase of the 2.4 GHz wireless interface is stored in plaintext form by the app in the datadatacom.dlink.dlinkwifishared_prefsROUTER_LIST.xml file.
- *Linksys*—For some imperceptible reason, this app was unable to discover the AP in the Wireless LAN network; note that the app uses the Internet Group Management Protocol (IGMP) to identify the AP. Therefore, to bind the AP with the app, we manually created an account. The communication between the app and the AP is done over HTTP. It was perceived that the datadatacom.cisco.connect.cloudapp_webviewLocal_Storageleveldb000005.ldb and datadatacom.cisco.connect.cloudapp_webviewLocal_Storageleveldb000026.ldb files contain in plaintext form both the 2.4 and 5 GHz Wi-Fi passphrases, with the former to also include the user’s email address. Another app file that stores the user’s email address in plaintext is the datadatacom.cisco.connect.cloudapp_webviewLocal_Storageleveldb000024.log. The *auth token* of the app (it is used to authenticate the user against the back-end) is stored in a JSON file; however, there is an expiration time of 7 days. No less important, the email address of the user can be also exposed through the *Logcat* command-line tool. Recall that *Logcat* can be executed either as an *adb* command or directly in a shell prompt of the emulator or connected device.
- *Mi WiFi*—It was observed that this app dumps sensitive information in the *Logcat* output. The exposed data include the session key the app uses to authenticate itself against the AP and the Wi-Fi passphrases for both the 2.4 and 5 GHz bands, all in plaintext form.
- *Netgear Nighthawk*—This app required the user to first create an account. Next, the app scans the wireless LAN network for a supported device; scanning is done over HTTP. After a device is found, the app prompts the user to enter their credentials for connecting to the AP’s web management interface. The analysis performed shows that the app holds in the datadatacom.netgear.netgearupshared_prefsDataModelLocalStorage.xml file the Wi-Fi passphrases for both bands in a plaintext form. Moreover, the “datadatacom.netgear.netgearupshared_prefsLAST_SESSION_PREFERENCE.xml” file includes the email address of the user. Through *Logcat*, the app exposes the Wi-Fi passphrases of both bands and the administrator’s username. Last but not least, the datadatacom.netgear.netgearupdatabasesnetgear_up_database.db database file stores the details of any device associated with the AP in the past. These pieces of data include MAC addresses in a plaintext form.
- *TP-Link Tether*—For using this app, the creation of a cloud-based user account is required. The app-to-AP communication is done over TLS v1.3 (cloud) and SSH v2 (AP). The analysis revealed that the datadatacom.tplink.tetherdatabasesgoogle_app_measurement.db database contains the Wi-Fi passphrases of both bands in plaintext form. The same situation regarding credential leakage is applicable to the datadatacom.tplink.tetherfiles.com.google.firebase.crashlyticslog-filescrashlytics-userlog-610FCD100211-0001-0C1D-A212A9CE2A17.temp temporary file the app creates. Moreover, the TP_TETHER_CACHE.DB database stores an MD5 hash of the administrator’s password. The *auth token* used to authenticate the app against the cloud service is stored in a JSON file in plaintext form; there is an expiration time of 7 days.

### 5.2. Smart TV

The LGThinQ app cannot be executed in rooted devices. For this reason, only the RemoteNOW one was analyzed, without however connecting it to an IoT device. It was perceived that this app uses Simple Service Discovery Protocol (SSDP) to identify compatible devices. The MD5 obsolete hashing scheme is used to store data in the app’s database.

### 5.3. Wireless IP Cameras

For dynamically analyzing this category of apps, we employed 3 modern wireless IP cameras, namely, Mi Home Security Camera 1080p, DLink DCS-6500LH, and TP-Link Tapo C200.

- *Mi Home*—This app was created with the *React Native* open-source UI software framework, but in this case, the app’s *React* package file is left unprotected. This package file contains all dependencies, along with their versions, *React* needs during compilation time. As a result, one is able to observe every dependency the app uses by just reading the datadatacom.xiaomi.smarthomefilespluginrnsdk10058androidrawnode_modules_reactnative_package.json file, and subsequently searching for any open CVE IDs to exploit it. Another important remark is that the datadatacom.xiaomi.smarthomedatabasesmiio.dbdatabaseand datadatacom.xiaomi.smarthomeshared_prefspassport_ui.xml files contain the user’s email address in plaintext.
- *mydlink*—This app keeps the user’s email address in the datadatacom.dlink.mydlinkunifieddatabasesmyDB file. Furthermore, the app stores the *auth token* in a JSON file that expires after 7 days.
- *TP-Link Tapo*—This app stores the camera’s MAC address in the datadatacom.tplink.iotshared_prefsaria_sp.xml file. Moreover, the *auth token* value is kept in a JSON file, with the relevant token to expire after 7 days.

### 5.4. Smart Wearable

All apps in this subsection details on apps was connected to any real-life IoT device; recall that *Genymotion* is unable to connect through a Bluetooth link.

- *Garmin Connect*—Our analysis showed that the datadatacom.garmin.android.apps.connectmobiledatabasescache-database and datadatacom.garmin.android.apps.connectmobiledatabasesgcm_cache.db files contain several sensitive pieces of information in plaintext form, including activity and health data, say, completed steps, burned calories, etc. As with other apps, the app’s *auth token* is stored in a JSON file, which expires after a week.
- *Fitbit*—It stores an assortment of plaintext sensitive data in different files. First, the email address of the user is stored in the datadatacom.fitbit.FitbitMobileshared_prefsApplicationSavedState.xml file. The datadatacom.fitbit.FitbitMobileshared_prefsprofile_lite.prefs.xml file reveals the username. The app’s sleep pattern is kept in the datadatacom.fitbit.FitbitMobileshared_prefsSleepSavedState.xml file, while the datadatacom.fitbit.FitbitMobilecachedatacachehttps3A2F2Fstatic0.fitbit.com2Fcontent2Fassets2Fsurvey2Fbc6fb5fe-36b8-4aec-be31-8bc34566e54e2Fsurvey_en.json file includes among others the woman’s birth control options along with any menstruation cycle data. Numerous sensitive pieces of information are stored in the app’s database.
- *Huawei Health*—It keeps all data in encrypted format. Interestingly, after purchase, for being functional, the watch must be paired to the Huawei Health app. Strangely, however, the current version of this app in the Google Play Store is obsolete. Therefore, the user needs to first download the so-called *AppGallery* app (the latter is a third-party app store managed by Huawei), which in turn provides access to the latest version of the Huawei Health app.
- *Mi Fit*—This app keeps the user’s personal goals, say, steps, calories, etc., in its database in cleatext form. The datadatacom.xiaomi.hm.healthshared_prefskeeper.xml file stores a number of sensitive information, including the current weight of the user in plaintext form. Lastly, sensitive data related to a female user, such as the last menstruation time, are kept in the datadatacom.xiaomi.hm.healthshared_prefsFemaleHealth_3077573711.xml file.
- *Samsung Health*—Apart from Samsung products, this app can be used to manage assorted IoT devices. For this reason, it is able to communicate with other similar apps, such as Fitbit. For activating the app, the user is required to create an account. The app mandates two-factor user authentication through the provision of a mobile phone number. Several files created by this app, including datadatacom.sec.android.app.shealthshared_prefsblood_glucose_trend_data_pref_sync_file_name.xml, contain sensitive user information in plaintext, such as the recorded user’s glucose level, their sleep condition, and food consumption. The app’s *auth token*, with an expiration time of 7 days, is also stored in the datadatacom.sec.android.app.shealthfilesPersistedInstallation.W0RFRkFVTFRdMTo3OTU2MjEzMTc4OTc6YW5kcm9pZDozYWRjOTkyNjJhYWIxNjI4.json file.

### 5.5. Smart Assistant

For this category of apps, we employed the Amazon Echo Dot (3rd generation) Smart hub, Samsung Dryer DV90N62632W, and the Google Nest Mini 2nd generation IoT devices along with the Amazon Alexa, Samsung SmartThings, and Google Home apps, respectively.

- *Amazon Alexa*—As already pointed out, for dynamically analyzing this app, the *Amazon Echo Dot* was utilized. To do so, and prior to the Alexa app’s installation, as a prerequisite, the Google Play Store app and Genymotion’s *OpenGApps* utility were installed in the emulator. Amazon Alexa requires an active Bluetooth connection to achieve the initialization of the Echo Dot device. However, as already mentioned, due to the incompatibility of *Genymotion* to provide such a connectivity interface, the initial setup of the Smart hub was accomplished with the aid of a physical device. Amazon Alexa also allows the user to sign in to an existing Amazon account, and this feature was exploited to connect to the device through the app installed in the emulator. The analysis revealed that this app keeps the user’s email address in the datadatacom.amazon.dee.appdatabasescom.google.android.datatransport.event file. Even more, the *auth token* value is kept in a JSON file, with the token to expire after 7 days.
- *Google Home*—The Google Nest Mini device was utilized when dynamically analyzing this app. The Google Play Store app was requested to exist in the user’s smartphone from this app. To do so, the *Open GAPPS* project offered from Genymotion was utilized. Then, to transfer music files from the smartphone to the Google Nest Mini, we installed Spotify for streaming music to the device. This was done with the aid of Miracast over Google Home. The communication takes place over TCP, UDP, MDNS, AJP13, and TLS v1.2 protocols. A handful of app files and two databases contain the user’s email address in plaintext. The server’s session token (Google Nest) was stored in an XML file, expiring after 3.5 h. Lastly, the *auth token* of the app is stored in the datadatacom.google.android.apps.chromecast.appfilesPersistedInstallation.W0RFRkFVTFRdMTo0OTg1Nzk2MzM1MTQ6YW5kcm9pZDpjNTMzYTJlZjUyMGNjZWM5.json file. The latter token expires after 7 days.
- *Samsung SmartThings*—The dynamic analysis of this app was accomplished with the use of the Samsung Dryer DV90N62632W. For connecting to the dryer, this app requires the user to login to their Samsung account. Communication is established over the TLSv1.2 protocol. The analysis revealed three distinct database files, namely datadatacom.samsung.android.oneconnectdatabasesCommonData.db, datadatacom.samsung.android.oneconnectdatabasesInternalSettings.db and datadatacom.samsung.android.oneconnectdatabasesMobileAsThing.db containing user’s sensitive information in cleartext. Among these pieces of information were the user’s email address, the Universally Unique Identifier (UUID) of the device, the user’s name and surname, their date of birth, sex, and time/date of each session. Cloud server access and refresh tokens were stored in a datadatacom.samsung.android.oneconnectdatabasesInternalSettings.db file, expiring after 21 h. Lastly, the *auth token* of the app is stored in the datadatacom.samsung.android.oneconnectsharedprefscom.google.android.gms.appid.xml file. As with all the other apps, this token expires after 7 days.

### 5.6. Smart Bulb/Plug

For this last IoT category, we employed a Smart LED Bulb 1S (color) along with the *Yeelight* app. For the Wipro app, no IoT device was used.

- *Yeelight*—It was observed that this app establishes a TCP connection to transfer the relevant data. This app requires the user to login with a Xiaomi user account. Only the datadatacom.yeelight.cherryshared_prefsmiot.xml XML file contains sensitive data, including the device’s MAC address and its firmware version. The app’s *auth token* is stored in a JSON file, which expires after a week.
- *Wipro*—Most of the data communicated or stored by this app are in encrypted form. The only sensitive data exposed is an *auth token* stored in the datadatawipro.comfilesPersistedInstallation.W0RFRkFVTFRdMToxNzM1MTYyMTA4NDA6YW5kcm9pZDoxMDAyNjZmYmNiYWViNzE3.json JSON file, expiring after 7 days.

### 5.7. Discussion

The rightmost 6 columns of Table 6 summarize the results obtained through dynamic analysis. Focusing on the IoT categories, it seems that the AP one is the most susceptible to misconfigurations and data leakages. As observed from the table, all the examined apps of this category were identified to be susceptible to at least one issue in regard to sensitive information leakage. Precisely, 5 apps of this kind were found to store sensitive information in plaintext form, including Wi-Fi passphrases and email addresses, while 3 of them exposed private information either through cleartext traffic, via the *Logcat* command-line tool, or both. It should be noted that Wi-Fi passphrase leakages is of major significance, given that many users tend to preserve for long periods of time the same passphrase(s) in their wireless routers [39].

The dynamic analysis also revealed leakages of sensitive information for all but one of the remaining IoT categories. That is, more than 50% of the apps across all the categories were found to potentially leak user’s sensitive information. Additionally, 45% of the examined apps (or 9 apps out of 20) were found to store the app’s *auth token* to an XML or JSON type of file. In all these cases, the expiration time of the token never exceeded the 7 days; this time window seems to be the standard for this type of apps. File leakage and obsolete hashing algorithm issues seem rather scanty, with only 3 apps in each of the first 3 IoT categories identified as vulnerable to them. Overall, under the reservation of the single app of this kind examined, the less affected category is the Smart TV, revealing only one issue pertaining to the use of the MD5 algorithm.

## 6. Related Work

There is a significant mass of works addressing vulnerability discovery and analysis in the IoT ecosystem. The survey article in [40] focuses on the attack surface of IoT devices and the available techniques to track down vulnerabilities. The authors discuss at a rather high level Web app issues, including SQL injection and XSS attacks. They also elaborate on a triad of challenges and opportunities, and provide discussions on future research directions. No experimental evaluation of IoT apps or devices are given in the context of that work. Moreover, the survey work in [41] explores the security challenges in the IoT domain, and examines typical security mechanisms used in the context of mainstream IoT communication protocols like ZigBee, Bluetooth Low Energy (BLE), and others [42]. Based on the literature, the authors also discuss several attacks exercised against real-life IoT devices. Lastly, they examine the security features of IoT devices’ microcontrollers used for connectivity purposes.

A couple of Android vulnerability analysis schemes targeting IoT devices have been given in [43,44]. The first, contributes a platform that speeds up vulnerability detection and analysis. This platform does not require the presence of actual devices or firmware. The authors elaborate on potentially vulnerable components, such as backend services, device rebranding, and others, commonly found in smart home IoT devices. They present results stemming from a plethora of devices from assorted vendors, but the apps analyzed by the authors were not popular. In the second, the authors analyzed the code of around 30 mobile apps that accompany IoT devices. Their goal was to figure out the way these apps communicate with the devices, and subsequently the identification of security issues. Their results showed the approximately 50% of the apps did not use strong encryption schemes. They also verified the results based on a handful of real-life devices. This study and its findings were mostly concentrated on network-oriented vulnerability issues, including cleartext HTTP traffic.

A more low-level vulnerability discovery and analysis approach, also assisted by a fuzzer called *IOTFUZZER*, has been contributed in [45]. The focus of this work is on discovering memory corruption vulnerabilities in IoT devices without having access to their firmware binaries. Once again, the analysis was based on the companion apps of such devices, and capitalized on the fact that such apps typically contain copious information regarding the app-to-device communication protocol. By examining (probing) nearly 20 IoT devices through the use of *IOTFUZZER*, the authors identified several memory corruption vulnerabilities, including buffer overflow and null pointer dereference.

The recent work in [46] proposed a both blockchain and deep learning based framework to improve the security level of Android IoT devices. Precisely, the authors highlight that their scheme is able to detect malware activities in real-time. This contribution also offers a special smart contract to discern malicious apps through the blockchain framework. That is, malware detection relies on a significant mass of static and dynamic features extracted from both malware and benign apps and subsequently used to train a deep learning model.

Overall, while significant, the existing work on this contemporary topic mostly focuses on three angles:

- Survey works addressing vulnerability discovery and analysis in the IoT devices realm [40,41]; naturally, such works do not provide new experimental results stemming from IoT devices or the associated apps.
- The examination of individual or a limited set of security issues of IoT devices [43] or their accompanying apps [44]. For devices, the focus is on potentially vulnerable components, while for apps, the concentration is on network-oriented vulnerability issues.
- The discovery of vulnerabilities through fuzz testing contacted against the IoT device [45] along with the accompanying app. Such works mostly target memory corruption vulnerabilities.

Based on the above, thus far the literature largely misses a full-fledged study concentrating solely on the security and privacy issues of the IoT device accompanying apps. The current paper aspires to fill this particular gap by:

- Furnishing a more holistic picture of this area of research. This is done by concentrating only on very popular companion apps, i.e., those that present more than 1M downloads, and by examining the apps from multiple prisms, namely both static and dynamic, and in an in-depth manner. Namely, the outcomes of our analysis originate from a diversity of static and dynamic features, the latter after pairing the app to real-life IoT devices. In this regard, the methodology and the results offered by this work address the specific issue from a more comprehensive angle.
- Embracing IoT apps belonging to the most prevailing categories of devices, thus allowing for the extrapolation of useful and insightful comparisons between them.

## 7. Conclusions

Focusing on app permissions, the Smart wearable category seems to gather the greater number of dangerous permissions per app. Precisely, 10 out of 12 apps in this category prompt the user for at least 10 permissions. This category also makes intense use of some permissions flagged as “Communication”. Naturally, this can be more or less justified by the nature of these type of apps. Overall, the use of location, utility, and storage type of permissions is apparent in all the categories of apps. This may be consistent with the purpose of the corresponding apps, but as explained in Section 3.2, exceptions do exist. For instance, it is mostly unclear why the permission relevant to the precise location of the user is necessary for a Smart TV or Smart bulb/plug app. On top of that, a general observation is that a significant number of apps across all categories make use of obsolete permissions, even those being superannuated since Android v5. Another pertinent issue is that the introduction of hard- and soft-restricted permissions by Android v10 cannot be effectively enforced if vendors use their own app stores, as in the case of Huawei. This is because permission *whitelisting* depends solely on the installer.

The results stemming from low-level static analysis reveal that a plethora of apps remain susceptible to even legacy weaknesses and vulnerabilities, including Janus. By just throwing a glance at Table 3, it can be easily perceived that almost all the discovered issues—Packers and CWEs 502, 919 are the only ones excluded—apply to the swarm of the apps. Again, this negative outcome makes rather clear that security is not of top priority in this ecosystem.

Roughly, the same unfavorable situation stands largely true when referring to app trackers. Only two apps remain free of trackers, while the rest incorporate several trackers of the same or different kinds. Moreover, the great mass of such trackers are particularly privacy-invasive, as they belong to the Analytics, Marketing, Identification, and Ads categories in a total that exceeds 75%.

Manifest analysis also exhibits several security misconfigurations and other relevant issues across all the categories of apps. Some of them such as the misconfigured *android:usesCleartextTraffic* flag, the use of alternative Launch modes, and the omission of permissions relevant to the Service, Broadcast, and Activity components are frequently met in apps irrespective of their category. This result also strengthens the above-mentioned inference regarding the security level of the different apps.

The results from shared library and outdated software component analysis are rather independent of the category of the app. Therefore, both these types of analysis can provide a universal picture regarding the security level of the various apps as a whole. From the one hand, the conclusion here is that some legacy exploit mitigation techniques relevant to shared libraries are not present in the great mass of apps; indicative of this situation is the high number of issues corresponding to *FORTIFY* and *RELRO*. On the other, although considerably fewer issues are observed in regard to outdated third-party software components, specific but critical libraries such as the OpenSSL one is found to be obsolete in about the one-third of the apps.

Regarding taint analysis, the apps in the Smart wearable category seem to overall accumulate the greatest number of issues; characteristically, 3 out of the 12 apps have been identified to have at least 700 potential issues, while 2 more at least 500. Besides, 2 apps in the same category were found to be susceptible to SQL injection attacks.

Unexpectedly, dynamic analysis designated the AP category as the one with the higher number of severe issues. All but one of the analyzed apps in this category store locally sensitive information, including the administrator’s credentials and Wi-Fi passphrases, in a plaintext form. Further, half of the apps in the same category use unencrypted (HTTP) links for app-to-AP’s web-based management interface communication. Data leaks related to *Auth token* is also a common issue for the Wireless IP cameras and Smart bulb/plug categories. Altogether, it can be said that the results obtained from dynamic analysis largely corroborate the general conclusion drawn from static analysis; app vendors and the relevant stakeholders should pay more attention to security and privacy.

All in all, if a security or privacy issue impacts an Android app, it is to a greater or lesser extent likely that it also directly or indirectly affects the IoT device itself. Putting it differently, the attack surface for an IoT device doubles due to the accompanying app; it does not matter if the device is secure, because a vulnerability in the associated app may provide the initial foothold for the attacker to, say, gain unauthorized access to the device. Indeed, some of the issues identified in the context of this work, e.g., transmission of cleartext traffic, outdated software components, the omission of protection against reverse engineering, and others, can eventually lead to compromising the IoT device.

It would be interesting to examine in a future work the impact of the recently introduced Manufacturer Usage Description Specification (MUD) standard [47] within the companion apps explored in the work. According to this standard, manufacturers, i.e., some entity along the supply chain, explicitly prescribes in a MUD reference profile the exact behaviors allowed for their devices according to the device’s precise functionality; the gist here is that each IoT device is supposed to serve a limited purpose, and therefore the device’s MUD profile is a set of rules and expected behaviors. For instance, a light bulb is intended to light a room. It may also be remotely controlled over the network, typically through a smartphone app. A MUD profile is obtainable by local network management systems through a URL. This allows updating the network (security) policies regarding the device.

Therefore, in the light bulb example, the local network management system will be aware that this device is not supposed to talk to other things, say, the air-conditioner or the printer. It is not also prescribed to interact with social networking sites. In this sense, the network is enabled to provide an extra protection layer to the device. All in all, while (as expected) none of the examined devices in the context of this work is MUD compatible, a future research avenue is to explore if and to what degree the introduction of MUD will also positively affect the security and privacy level of the mobile apps that go hand and glove with this kind of devices.

A similar research direction can be followed with respect to the Device Identifier Composition Engine (DICE) industry standard (a suite of hardware and software based techniques that aims in certifying the health of software and hardware on IoT devices) and the Matter smart home interoperability protocol, which is developed with the promise to focus on security by design.

## Figures and Tables

**Table 1:** Official management apps of popular IoT devices.

App Name	Examined ver.	Popularity	Connectivity
AP			
ASUS Router	2.0.0.6.28	1M+	Wi-Fi
D-Link WiFi	1.4.4 build 1	1M+	Wi-Fi
Huawei HiLink	9.0.1.323	10M+	Wi-Fi
Linksys	2.16.1	1M+	Wi-Fi
MikroTik Pro	1.3.20	1M+	Wi-Fi
Mi Wi-Fi	4.2.9	1M+	Wi-Fi
Netgear Nighthawk	2.11.5.1716	1M+	Wi-Fi
TP-Link Tether	3.4.28	10M+	Wi-Fi
UniFi Network	3.9.3	1M+	Wi-Fi
Smart TV			
LG ThinQ	3.6.12110	10M+	Wi-Fi
RemoteNOW	5.01.011	1M+	Wi-Fi
Wireless IP cameras			
Arlo	3.5.4_28395	1M+	Wi-Fi
HiLookVision	3.10.1.0924	5M+	Wi-Fi
iVMS-4500	4.7.12	10M+	Wi-Fi
Mi Home	6.10.709	10M+	Wi-Fi
mydlink	2.5.0	1M+	Wi-Fi
mydlink Lite	3.8.14	1M+	Wi-Fi
Nest	5.66.0.7	5M+	Wi-Fi
TP-Link Tapo	2.4.25	1M+	Wi-Fi
Wyze	2.24.23	1M+	Wi-Fi
Smart wearable			
Galaxy Wearable (Samsung Gear)	2.2.17082261	500M+	Bluetooth
Garmin Connect	4.47	10M+	Bluetooth
Fitbit	3.18	50M+	Bluetooth
Huawei Health	12.0.8.300	100M+	Bluetooth
Mi Fit	5.3.2	50M+	Bluetooth
Polar Beat: Running & Fitness	3.5.2	1M+	Bluetooth
Polar Flow – Sync & Analyze	6.6.0	5M+	Bluetooth
realme Link	1.2.480.53	1M+	Bluetooth
Samsung Health	6.18.8.005	1B+	Bluetooth
Suunto	4.45.2	1M+	Bluetooth
Withings Health Mate	5.6.1	1M+	Bluetooth
Zepp	6.1.4-play	5M+	Bluetooth
Smart assistant			
Amazon Alexa	2.2.422256.0	50M+	Wi-Fi/Bluetooth
Google Home	2.42.1.14	100M+	Wi-Fi/Bluetooth
Gosund - include NiteBird	4.4.3	1M+	Wi-Fi/Bluetooth
MyFRITZ!App	2.17.3 (16362) Beta	1M+	Wi-Fi
Samsung SmartThings	1.7.70.21	500M+	Wi-Fi/Bluetooth
Smart bulb/plug			
Philips Hue	4.4.0	5M+	Wi-Fi/Bluetooth
Yeelight	3.3.06	1M+	Wi-Fi/Bluetooth
Wiz	1.23.1	1M+	Wi-Fi/Bluetooth
Wipro Next Smart Home	3.22.0	1M+	Wi-Fi/Bluetooth

**Table 2:** Identified permissions per examined app.

App	Utility				Authentication				Location				Storage				Phone										Communication									
	U1	U2	U3	U4	A1	A2	A3	A4	L1	L2	L3	L4	S1	S2	S3	S4	P1	P2	P3	P4	P5	P6	P7	P8	P9	P10	C1	C2	C3	C4	C5	C6	C7	C8	C9	Total
AP																																				
ASUS Router	+								+	+			+	+	+							+														7
D-Link WiFi	+								+									+																		3
Huawei HiLink	+				+		+	+	+	+			+	+	+							+	+													11
Linksys	+						+		+	+				+																						5
Mikrotik Pro													+	+																						2
Mi Wi-Fi	+				+	+	+	+	+	+			+	+			+	+				+	+													13
Netgear Nighthawk	+	+					+		+	+			+	+			+																			8
TP-Link Tether	+								+	+			+	+																						5
UniFi Network	+								+				+	+																						4
Smart TV																																				
LG ThinQ	+	+				+	+	+	+	+	+		+	+			+		+	+																13
RemoteNOW		+					+		+	+			+	+		+	+					+	+													10
Wireless IP cameras																																				
Arlo	+	+					+		+	+	+		+	+				+	+														+			11
HiLookVision	+	+							+	+			+	+			+	+					+													9
iVMS-4500	+	+							+	+			+	+	+		+	+				+	+													11
Mi Home	+	+			+	+	+	+	+	+			+	+	+	+	+	+					+	+												16
mydlink	+	+							+		+		+	+			+	+	+													+				10
mydlink lite	+	+							+	+			+	+				+																		7
Nest	+	+					+		+		+		+	+					+																	8
TP-Link Tapo	+	+							+	+			+	+			+																			7
Wyze	+	+			+				+	+	+		+	+		+	+	+	+				+				+	+			+	+		+		18
Smart wearable																																				
Galaxy Wearable							+		+					+			+		+	+		+	+								+					9
Garmin Connect	+					+	+	+	+	+	+		+	+			+	+	+						+				+		+	+		+		17
Fitbit	+	+					+		+	+			+	+	+		+		+						+	+			+		+	+		+		16
Huawei Health	+	+			+				+	+	+		+	+	+	+	+		+			+	+	+		+		+	+		+	+		+	+	22
Mi Fit	+	+		+			+		+	+	+		+	+	+		+		+				+						+		+	+		+		17
Polar Beat				+			+		+	+	+			+																						6
Polar Flow				+			+		+	+	+		+	+	+		+		+						+	+					+					13
realme Link	+	+	+	+					+	+	+		+	+	+		+		+				+	+				+			+	+		+		18
Samsung Health	+	+		+		+	+		+				+	+			+		+		+	+														12
Suunto				+					+	+	+		+	+			+		+				+								+					10
Withings Health Mate	+			+			+		+	+	+			+	+		+		+												+					11
Zepp	+	+		+			+		+	+	+		+	+	+		+		+			+	+						+		+	+		+		18
Smart assistant																																				
Amazon Alexa	+	+		+	+	+	+	+	+	+	+	+	+	+			+		+								+	+	+	+		+		+		21
Google Home	+	+					+	+	+					+																		+				7
Gozund	+	+							+	+	+		+	+	+		+																			9
MyFRITZ!App																	+	+																		2
Samsung SmartThings	+	+					+		+	+	+		+	+			+		+		+	+		+												13
Smart bulb/plug																																				
Philips Hue									+	+	+																									3
Yeelight	+	+			+	+	+	+	+	+			+	+			+	+					+													13
Wiz					+				+	+			+	+																						5
Wipro	+	+							+	+			+	+																						6
TOTAL	32	24	1	9	8	7	22	8	39	32	18	1	33	38	12	4	26	12	18	2	2	10	14	4	3	3	2	4	6	1	11	10	+	8	+	–

**Table 3:** Identified weaknesses and other security issues per examined app. The •, ⊛, ⊠, ⊞, and ⊡ symbols in the third column denote “cleartext traffic to all domains”, “cleartext traffic for specific domains”, “trust system certificates”, “trust user installed certificates”, and “bypass certificate pinning”, respectively.

App	Janus	Network Security	APK Signing	Packers	89	250	276	295	312	327	330	502	532	649	749	919	Total
AP																	
ASUS Router	+	•			+		++	++	+	3	+		+				13
D-Link WiFi	+		+						+	3	+		+		+		9
Huawei HiLink	+		+		+		+	++	+	3	+		+	+	+		14
Linksys	+		+				++		+	3	+		+	+	+		12
Mikrotik Pro	+				+		++		+	2	+		+				9
Mi Wi-Fi	+	•, ⊠	+		+		++		+	3	+		+	+			13
Netgear Nighthawk	+		+		+		++	+	+	3	+		+	+	+	+	15
TP-Link Tether	+	•	+		+		++	+	+	3	+		+	+	+		15
Unify Network	+		+		+		++		+	3	+		+				11
Smart TV																	
LGThinQ		•	+		+		++	++	+	3	+		+	+	+		15
RemoteNOW	+	•			+		+	++	+	2	+		+	+	+		13
Wireless IP cameras																	
Arlo	+				+	+	++	+	+	2	+		+	+	+	+	14
HiLookVision	+	•	+		+		++	++	+	3	+		+	+	+		16
iVMS-4500		•, ⊠			+		++	+	+	3	+		+	+	+	+	15
Mi Home	+	•, ⊠, ⊞	+	++	+		++	++	+	3	+		+	+	+	+	21
mydlink	+		+		+		++		+	3	+		+	+	+		13
mydlink lite	+		+		+		+	+	+	3	+		+				11
Nest	+				+		++		+	2	+		+	+			10
TP-Link Tapo	+	•	+		+		++		+	3	+		+	+	+	+	15
Wyze		•, ⊠			+		++	+	+	3	+		+	+	+	+	15
Smart wearable																	
Galaxy Wearable	+		+		+		+		+	1	+		+				18
Garmin Connect	+	⊛			+		++	+	+	2	+		+	+	+		13
Fitbit		⊛, ⊠, ⊞	+		+		++		+	2	+		+	+	+	+	15
Huawei Health	+		+	+	+		++	++	+	3	+		+	+	+	+	18
Mi Fit	+	•, ⊠	+	+	+		++	++	+	3	+		+	+	+		18
Polar Beat	+		+		+		+		+		+		+				17
Polar Flow	+		+		+		++		+	2	+		+	+		+	12
realme Link	+		+		+		++	+	+	3	+		+	+	+	+	15
Samsung Health	+		+		+		++		+	3	+		+	+	+	+	14
Suunto	+	⊛	+		+		++		+	2	+		+				11
Withings Health Mate			+		+		++		+	2	+		+	+			10
Zepp	+	⊛, ⊠, ⊞, ⊡	+	+	+		++	++	+	3	+		+	+	+	+	21
Smart assistant																	
Amazon Alexa	+				+		++	+	+	1	+	+	+		+		11
Google Home	+	•			+		++	+	+	2	+		+	+	+		13
Gosund	+	•			+		++		+	1	+		+	+	+		11
MyFRITZ!App	+		+		+		++		+	2	+		+	+			11
Samsung SmartThings	+	•, ⊠	+		+		++		+	3	+		+	+	+	+	18
Smart bulb/plug																	
Philips Hue	+		+		+		++		+	2	+		+	+			11
Yeelight	+				+		+		+	3	+		+	+	+	+	12
Wiz	+	•, ⊛, ⊠			+		++		+	2	+		+		+	+	14
Wipro	+	•			+		++		+	3	+		+	+	+	+	14
Total	36	20	27	4	39	1	40	19	41	40	41	1	41	31	27	16	–

**Table 4:** List of identified potential issues in the manifest file of each app.

App	Intent-Filter On			Intent-Filter Off			Content	Task	Launch	Cleartext	Backup	Priority	SMS
	Service	Broadcast	Activity	Service	Broadcast	Activity							
AP													
ASUS Router	1		1				1						
D-Link WiFi	2			1	1				1	+		+	
Huawei HiLink		4	2		1	1			2	+			
Linksys	2	1								+			
Mikrotik Pro				1		1			1		+		
Mi Wi-Fi		6	3	1	3	1			1				
Netgear Nighthawk	1	2	2	1				1		+			
TP-Link Tether	1		2						3				
UniFi Network			1							+	+		
Smart TV													
LG ThinQ	2	6	15	1	1	2							
RemoteNOW		4		8	2	3			6	+	+		
Wireless IP cameras													
Arlo	1	2	3		1	1		2					
HiLookVision									14				
iVMS-4500	2	6		4		1		3	2			+	
Mi Home	2	7	17	8	10	28	2	26	72			+	
mydlink	1		1	1	1	3				+			
mydlink lite	2		2	2									
Nest		3	2		1	2	1						
TP-Link Tapo			2										
Wyze		4	1		1	1							+
Smart wearable													
Galaxy Wearable		2	1		1		1		2			+	
Garmin Connect	1	3	11	5	1	5	5						
Fitbit	1	5	6		1	2	1						
Huawei Health	22	19	31								+		
Mi Fit		4	13	4	6	8	2	2	49			+	
Polar Beat			1		1								
Polar Flow		3	1	1		2							
realme Link	1			1		1	1					+	
Samsung Health	2	4	2	2	8	9	7	3		+		+	
Suunto	1	9	3	1		3							
Withings Health Mate	1	4	3	2	1	4	1						
Zepp	3	5	11	13	5	8	4	1				+	
Smart assistant													
Amazon Alexa	4	15	17	10	8	6	3	9					
Google Home					12	6		3		+	+		
Gozund		1	7			3				+			
MyFRITZ!App		12	10							+			
Samsung SmartThings	2	3	3	2	3	18	6	5					
Smart bulb/plug													
Philips Hue		3	1			1				+			
Yeelight			3	3	5	1	1	4	4		+		
Wiz		2	3	1		5			3				
Wipro	2	3	5			3			35				

**Table 5:** Outdated third-party software components per app. The greater the number of “+” signs, the more the number of obsolete components in this app.

App	SQLite	libjpeg	OpenSSL	jQuery	Python	libpng	OpenCV	libcurl	zlib	expat	FFmpeg	Total
AP												
ASUS Router			+									1
D-Link WiFi				+								1
Linksys				+								1
Mikrotik Pro	+		+									2
Mi Wi-Fi			+									1
Netgear Nighthawk					+		+					2
TP-Link Tether	+		+									2
Smart TV												
LGThinQ								+				1
RemoteNOW										+		1
Wireless IP cameras												
HiLookVision		+	++++			+						6
iVMS-4500			++									2
Mi Home	+	+	+++		+	++	+		+			10
mydlink			+	+								2
Wyze	+		+									2
Smart wearable												
Garmin Connect					+		+					2
Fitbit	+											1
Mi Fit		+	+		+		+					4
Polar Flow	+											1
Smart assistant												
Alexa	+	+	+									3
MyFRITZ!App			+									1
Samsung SmartThings	+											1
Smart bulb/plug												
Wiz			+									1
Wipro	+	+	++		+		+				+	7
Total	9	5	14	3	4	2	5	1	1	1	1	-

**Table 6:** Overview of issues identified through dynamic analysis. CSIF denotes the cleartext sensitive information, say, Wi-Fi passphrases kept in app’s files.

App	Tested Device	Cleartext Traffic	CSIF	Logcat Leak	Auth Token Leak	File Leak	Obsolete Alg.
AP							
ASUS Router	RT-AX88U	+	+				
D-Link WiFi	DIR-X1860		+				
Linksys	MR7350	+	+	+	+		
Mi Wifi	AX1800			+			
Netgear Nighthawk	RAX40	+	+	+			
TP-Link Tether	AX10v1		+		+		+
Smart TV							
RemoteNOW	–						+
Wireless IP cameras							
Mi Home	Sec. Cam. 1080p		+			+	
mydlink	DCS-6500LH		+		+		
TP-Link Tapo	C200		+		+		
Smart wearable							
Garmin Connect	–		+		+		
Fitbit	–		+				
Huawei Health	–						
Mi Fit	–		+				
Samsung Health	–		+		+		
Smart assistant							
Amazon Alexa	Amazon Echo Dot 3rd gen.		+		+		
Google Home	Google Nest Mini 2nd		+		+		
Samsung SmartThings	Samsung Dryer DV90N62632W		+		+		
Smart bulb/plug							
Yeelight	Smart LED Bulb 1S		+		+		
Wipro	–				+		

## Data Availability

All data are made available in the manuscript.

## Abbreviations

The following abbreviations are used in this manuscript:

ADB	Android Debug Bridge
AP	Access Point
API	Application Programming Interface
APK	Android Application Package
APKiD	Android Application Identifier
App	Application
APT	App Transport Security
BLE	Bluetooth Low Energy
CVE	Common Vulnerabilities and Exposures
CWE	Common Weakness Enumeration
CWSS	Common Weakness Scoring System
DEX	Dalvik Executable File
DoS	Denial of Service
ELF	Executable and Linkable Format binary
GOT	Global Offset Table
GUI	Graphical User Interface
HTTP(S)	Hypertext Transfer Protocol (Secure)
IGMP	Internet Group Management Protocol
IoT	Internet Of Things
IP	Internet Protocol
Jadx	Dex to Java decompiler
Json	JavaScript Object Notation
LAN	Local Access Network
LLDP	Link Layer Discovery Protocol
MitM	Man-in-the-Middle
MobSF	Mobile Security Framework
MMS	Multimedia Messaging Service
MUD	Manufacturer Usage Description
NDK	Android Native Development Kit
NX	No-Execute
OWASP	Open Web Application Security Project
RELRO	Relocation Read-Only
RPATH	Run-Time Search Path
SaaS	Software-as-a-Service
SOHO	Small Office Home Office
SMS	Short Message Service
TLS	Transport Layer Security
VM	Virtual Machine
XML	Extensible Markup Language
XSS	Cross-Site Scripting

## Appendix A

### Appendix A.1. List of Identified Trackers per App

- ASUS Router: Google Firebase.
- D-Link WiFi: Google Firebase, CrashLytics.
- MyFRITZ!App: Google Firebase, CrashLytics.
- Huawei HiLink: Huawei (HMS) Core.
- Linksys: Google Firebase, CrashLytics, Splunk MINT.
- Mikrotik Pro: Google Firebase, CrashLytics.
- Mi Wifi: Tencent.
- Netgear Nighthawk: Google Firebase, Google Analytics, Google Tag Manager, CrashLytics, Instabug, Optimizely, Urbanairship.
- TP-Link Tether: Google Firebase, CrashLytics.
- Unify: Google Firebase, CrashLytics, New Relic.
- LGThinQ: Adobe Experience Cloud, Dynatrace, Google Firebase, Keen, CrashLytics, Facebook Login, Facebook Places, Facebook Share, Google AdMob, Salesforce Marketing Cloud, Treasure Data.
- Arlo: Google Firebase, CrashLytics, Swrve.
- HiLookVision: Bugly, Google Firebase.
- iVMS-4500: Flurry, Huawei (HMS) Core.
- Mi Home: AutoNavi, Bugly, Facebook Login, Facebook Analytics, Facebook Places, Facebook Share, Huawei (HMS) Core, Segment, Tencent.
- mydlink: Google Firebase, CrashLytics, Facebook Login.
- Wyze: Braze, Google Firebase, CrashLytics, Segment.
- Garmin Connect: Google Firebase, CrashLytics, Facebook Login, Facebook Share.
- Fitbit: Google Firebase, CrashLytics, Facebook Login, Facebook Analytics, Facebook Places, Facebook Share, MS Visual Studio Analytics, MS Visual Studio Crashes, MixPanel, Optimizely, Salesforce Marketing Cloud.
- Huawei Health: AutoNavi, Huawei (HMS) Core.
- Mi Fit: AutoNavi, Facebook Login, Facebook Places, Facebook Share.
- Polar Beat: Google Firebase, CrashLytics, Google AdMob.
- Polar Flow: Google Firebase, Google Analytics, Google Tag Manager, CrashLytics, Huawei (HMS) Core.
- realme Link: Google Firebase, CrashLytics, Facebook Login, Facebook Analytics, Facebook Share.
- Samsung Health: AutoNavi, Google Firebase, Google Analytics, Google Tag Manager.
- Suunto: Braze, Google Firebase, CrashLytics, Facebook Login, Facebook Share, HelpShift, Mapbox.
- Withings Health Mate: Google Firebase, CrashLytics, Huawei (HMS) Core.
- Zepp: AutoNavi, Facebook Login, Facebook Analytics, Facebook Share.
- Amazon Alexa: Amazon Ads, Amazon Analytics, Bugsnag, Google Firebase, Metrics.
- Google Home: Google Firebase, Google Analytics.
- Gosund: Google Firebase, Flurry, CrashLytics, Facebook Login, Facebook Analytics, Facebook Places, Facebook Share.
- Samsung SmartThings: MS Visual Studio Analytics, MS Visual Studio Crashes.
- Philips Hue: Braze, Google Firebase, CrashLytics.
- Yeelight: Google Firebase, CrashLytics.
- Wiz: Google Firebase, CrashLytics, Facebook Login, Facebook Analytics, Facebook Places, Facebook Share, HelpShift.
- Wipro: Google Firebase.

### Appendix A.2. List of Identified Outdated Dependencies per App

The following list contains every outdated dependency we found per device. The categorization that took place is based on each outdated version and the parenthesis occurs of how many open CVE IDs the relevant outdated version contains, separated on the severity level, i.e., medium (M), high (H), or critical (C).

- ASUS Router, OpenSSL 1.0.2h (10M/8H/3C).
- D-Link WiFi, jQuery 1.7.2 (1M).
- MyFRITZ!App, OpenSSL 1.0.2p (4M).
- Linksys, jQuery 3.0.0 (1H).
- Mikrotik Pro, SQLite 3.26.0 (2H/1M), and OpenSSL 1.1.1 (4M).
- Mi Wi-Fi, OpenSSL 1.0.1h (10M/8H/3C).
- Netgear Nighthawk, Python 2.7 (1M/2H/1C), and OpenCV 3.4.3 (2M/3H).
- TP-Link Tether, SQLite 3.28.0 (1M/1H), and OpenSSL 1.1.1b (1M).
- LGThinQ, libcurl 7.65.1 (1H/2C).
- RemoteNOW, expat 2.0.1 (1M/5H/1C).
- HiLookVision, Libjpeg 1.5.0 (1M/1H), and OpenSSL 1.0.1c (13M/13H/7C), 1.0.2k (6M), 1.0.2q (1M), and 1.0.2p (3M).
- iVMS-4500, OpenSSL 1.0.1c (13M/13H/7C), and 1.0.2k (6M).
- Mi Home, SQLite 3.28.0 (1M/1H), Libjpeg 1.5.0 (1M/1H), OpenSSL 1.1.1b (1M), 1.0.2k (6M), and 1.0.2n (3M), Python 2.7 (1M/2H/1C), libpng 1.6.22beta03 (1C), and 1.6.26 (1C), OpenCV 4.0.1 (2M/3H), zlib 1.2.8 (2H/2C).
- mydlink, OpenSSL 1.0.1g (10M/8H/3C).
- Wyze, SQLite 3.28.0 (1M/1H), OpenSSL 1.1.1b (1M).
- Garmin Connect, Python 2.7.12 (1M/2H/1C), and OpenCV 2.4.13 (2M/17H).
- Fitbit, SQLite 3.20.1 (1M/3H).
- Mi Fit, libjpeg 1.5.0 (1M/1H), and Python 2.7.12 (1M/2H/1C).
- Polar Flow, SQLite 3.31.0 (1M/1H).
- Amazon Alexa, SQLite 3.13.0 (1M/2H/1C).
- Samsung SmartThings, SQLite 3.8.11.1 (2M/2H/1C).
- Wiz, 1.0.2k (6M).
- Wipro, SQLite 3.31.0 (1M/1H), libjpeg 1.5.3 (1M/1H), OpenSSL 1.1.1g, and 1.1.1e (1M), Python 2.7.10 (2M/2H/2C), and 2.4.11 (2M/17H), ffmpeg 3.2.5 (11M/5H/1C).
